# Weather State Ants Optimizer: A Markov-Driven Variable-Structure Metaheuristic

**DOI:** 10.3390/biomimetics11090626

**Published:** 2026-09-02

**Authors:** Xiubo Xia, Jian Sun, Xiaoyu Geng, Pu Zhang, Yongling Fu

**Affiliations:** School of Mechanical Engineering and Automation, Beihang University, Beijing 100191, China; xiaxiubo@buaa.edu.cn (X.X.); buaasunjian@buaa.edu.cn (J.S.); zpungry@buaa.edu.cn (P.Z.)

**Keywords:** Weather State Ants Optimizer, metaheuristic algorithm, Markov process, dynamic variable structure, exploration-exploitation balance, swarm intelligence

## Abstract

Metaheuristics require sustained global search without sacrificing local refinement, yet many variable-structure methods change operators through one-way iteration schedules. We introduce the Weather State Ants Optimizer (WSAO), in which a discrete-time Markov chain recurrently selects one of three population updates. Sunny, cloudy, and rainy states correspond to global exploration, movement toward nests, and local refinement, respectively. An archive-based mechanism also maintains several spatially separated nests as concurrent search centers. Thirty independent runs compared WSAO with 11 algorithms on 29 CEC2017 and 12 CEC2022 functions. WSAO achieved the lowest Friedman mean rank on both suites, at 2.48 and 2.33. Across five constrained design cases, it joined the leading group by mean objective value on four cases and ranked second on pressure-vessel design. Targeted CEC2022 controls showed that no alternative transition matrix dominated the baseline. Eliminating the trial perturbation worsened every selected function, whereas the contribution of multiple nests depended on the landscape structure. The combined evidence supports recurrent state-controlled search as a competitive framework for continuous numerical and constrained optimization.

## 1. Introduction

Many engineering design tasks combine nonlinearity, multiple local optima, nondifferentiability, or discrete decisions. In such settings, an exact model or usable gradient may not be available. Reviews covering Internet-of-Things systems, network-on-chip design, and combinatorial applications document the resulting use of metaheuristic search [1,2,3]. These methods offer flexible population-based exploration with limited dependence on problem-specific derivatives [4,5].

A central design decision is how search effort is divided between discovering new regions and refining candidates already found [6,7]. Broad sampling improves coverage but can delay convergence. Intensive refinement accelerates local progress but can trap a population prematurely. Variable-structure methods assign different operators to different phases to manage this trade-off [8,9]. However, an iteration-triggered phase is usually left permanently once its scheduled interval ends. Such schedules do not support stochastic recurrence of an earlier population strategy.

Weather changes can reorganize collective foraging, migration, and social activity in animals [10,11,12]. WSAO translates this environmental responsiveness into three algorithmic states. At each generation, a transition matrix selects the next complete population update. Because the chain can revisit any state, a search structure remains available after the population has switched away from it.

Markov models have previously controlled numerical parameters and sequences of heuristic operators [13]. Here, the sampled state instead determines the update equation applied to the full population. This role separates WSAO from methods that vary one coefficient or advance through predetermined phases. A fixed matrix also makes the state dynamics reproducible without introducing an additional fitness-feedback controller.

The evaluation combines 41 functions from the 2017 and 2022 IEEE Congress on Evolutionary Computation benchmark suites (CEC2017 and CEC2022) with five constrained engineering problems. Eleven established and recent optimizers serve as comparators. Suite-level ordering uses Friedman mean ranks, while Holm-adjusted Wilcoxon rank-sum tests examine individual function contrasts.

The study makes four specific contributions:

1. A weather-state controller replaces a one-way schedule with recurrent probabilistic selection among three population-level search equations.
2. A dynamic nest archive converts spatially separated elite candidates into simultaneous centers for guided refinement.
3. Repeated comparisons against 11 algorithms cover 29 CEC2017 and 12 CEC2022 functions at D=10, with nonparametric statistical assessment.
4. Five constrained design cases test whether performance transfers beyond unconstrained numerical benchmarks.

Section 2 establishes the methodological context. Section 3 defines the controller, nests, and state-specific equations. Section 4 presents the numerical protocol and results, followed by the constrained cases in Section 5. Section 6 and Section 7 interpret the evidence and delimit the claims.

## 2. Related Work

### 2.1. Metaheuristic Algorithms and Exploration–Exploitation Balance

Exploration and exploitation are implemented through many algorithmic traditions [6]. Genetic Algorithms and Differential Evolution evolve candidate sets through selection and variation [14,15]. Particle Swarm Optimization (PSO), Ant Colony Optimization, and Artificial Bee Colony instead model distributed collective search [16,17,18]. Other families include physics-inspired methods such as Simulated Annealing and Gravitational Search [19,20], and human-inspired schemes such as Teaching–Learning-Based Optimization and Imperialist Competitive Algorithm [21,22].

More recent optimizers draw operators from honey-badger foraging, jellyfish motion, cuckoo reproduction, porcupine defense, and planetary motion [23,24,25,26,27]. A biological or physical metaphor alone does not establish algorithmic novelty. Critical analyses therefore recommend separating the implemented mechanism from its narrative inspiration [28,29]. WSAO follows that distinction by defining each state through an explicit update equation.

Operator and parameter adaptation provide another development path. Differential Evolution variants have introduced self-adaptation, external archives, success-history memories, population-size reduction, Covariance Matrix Adaptation Evolution Strategy (CMA-ES) hybridization, and sinusoidal parameter ensembles [30,31,32,33,34,35]. Success-History Based Adaptive Differential Evolution with Linear Population Size Reduction (LSHADE) and its ensemble sinusoidal adaptation variant (LSHADE-EpSin, where “EpSin” denotes the ensemble sinusoidal parameter adaptation) are therefore included as strong non-metaphorical baselines for continuous optimization.

Machine-learning-assisted metaheuristics extend online adaptation by learning parameter or operator choices from search feedback. For example, Wang et al. combined Q-learning with Differential Evolution to adjust the scaling factor and crossover rate while selecting mutation strategies during a run [36]. WSAO follows a different design: its fixed Markov matrix selects complete population-level update equations without online reward learning or policy training.

### 2.2. Engineering Applications of Metaheuristics

Engineering uses of metaheuristics range from machining and scheduling to sensor placement, communication networks, water-quality calibration, and medical imaging [4,5,37,38,39,40,41,42]. Evaluation must nevertheless reflect the application. Molecular-geometry studies use structural descriptors, whereas constrained optimal-power-flow studies jointly assess Pareto quality and feasibility [43,44]. These examples motivate the constrained design tests used here. They also require explicit formulations, identical constraint treatment, and matched computational budgets.

### 2.3. Variable-Structure and Adaptive Metaheuristics

Dynamic search allocation can be organized into three broad mechanisms:

1. **Population partitioning** assigns different roles to subgroups. Grey Wolf Optimizer uses a hierarchy, while Artificial Bee Colony separates employed, onlooker, and scout behaviors [45,46]. Fixed roles may misallocate effort when the required search balance changes.
2. **Parameter scheduling** changes coefficients without replacing the full update structure. Examples include decreasing inertia in PSO variants and the scheduled parameter *a* in WOA [47,48].
3. **Phase-dependent structures** apply distinct models during predetermined intervals. Marine Predators Algorithm (MPA) uses three iteration ranges, while the Sinh Cosh Optimizer (SCHO) changes between trigonometric search modes [8,9]. These schedules do not revisit an earlier phase stochastically.

### 2.4. Markov Processes in Optimization

Markov-based heuristic control appears at several levels. A Markov-chain hyper-heuristic treats low-level heuristics as states and learns transition preferences online [49]. Conditional Markov Chain Search composes search components through explicit transitions that may depend on the preceding outcome [50]. Nested chains have also selected global and local operator sequences for continuous problems [51]. Other decision models adjust numerical parameters during the run [13]. These studies control operator sequences or coefficients. WSAO instead applies one fixed three-state chain to choose the next full-population equation. It requires neither online transition learning nor a one-way phase schedule.

### 2.5. Positioning of WSAO

Table 1 compares representative methods using four structural criteria relevant to the proposed controller.

The columns distinguish structural variation, probabilistic selection, concurrent search centers, and the source of switching. Within this set, WSAO uniquely combines recurrent Markov transitions with multiple active centers. The comparison concerns mechanism only and does not rank empirical performance.

## 3. Proposed Algorithm: Weather State Ants Optimizer

### 3.1. Inspiration

Weather can change both the range and organization of collective animal activity [10,11,12]. Rain suppresses leaf-cutting-ant foraging, flight conditions alter dragonfly migration, and common waxbills reorganize group behavior as weather changes. These observations motivate environmental state changes as triggers for population-level search behavior.

WSAO converts this inspiration into three deliberately abstract regimes:

- **Sunny**: widespread foraging corresponds to population-wide exploration.
- **Cloudy**: collective relocation corresponds to movement toward candidate nests.
- **Rainy**: activity concentrated near nests corresponds to local refinement [10].

The mapping is algorithmic rather than a quantitative ecological model. Each weather label identifies one complementary search equation. A discrete-time Markov chain governs their order, allowing a previously used strategy to return at a later generation.

**Figure 1:**
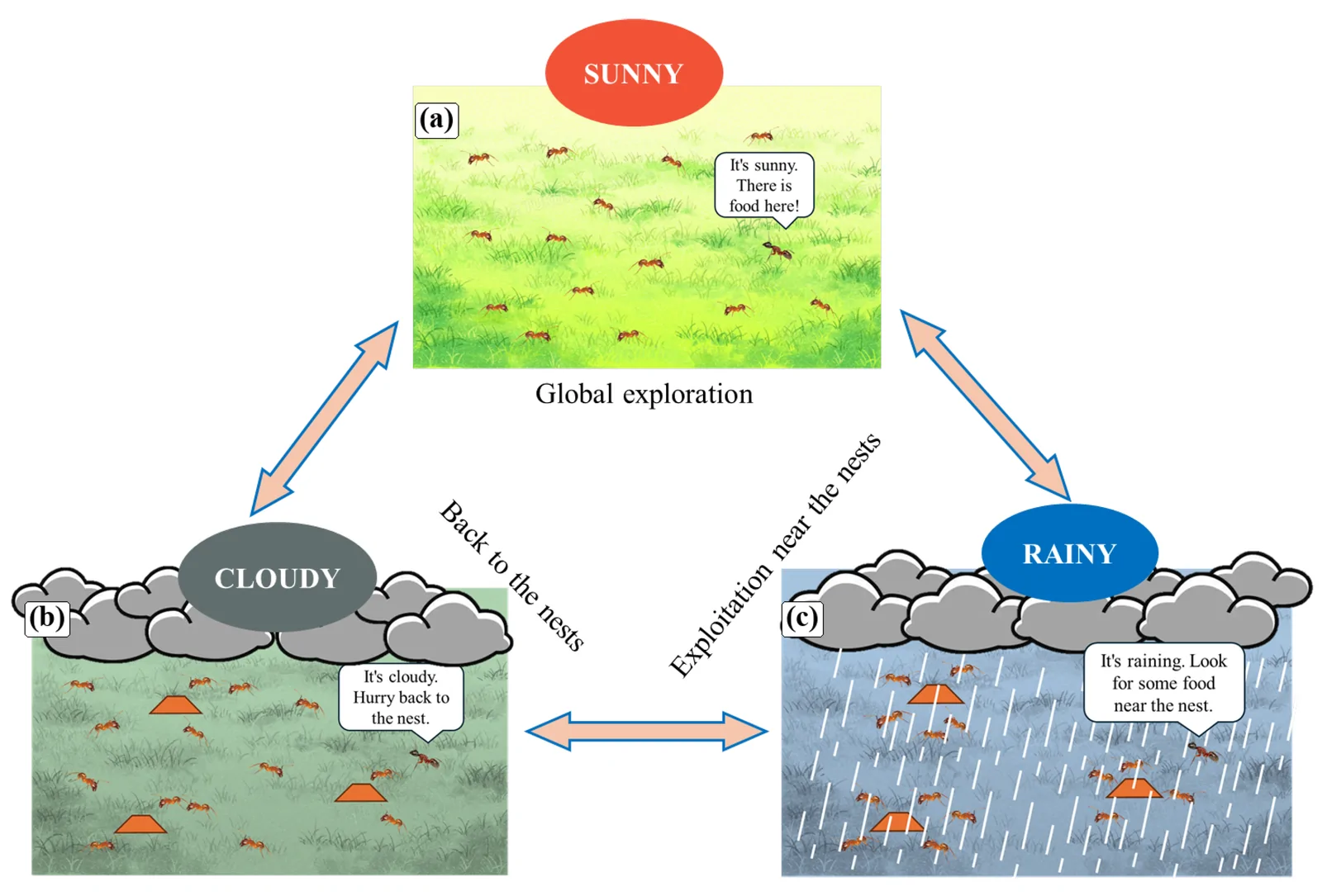
Behavior-to-search correspondence in WSAO: (**a**) sunny conditions motivate wide-ranging exploration, (**b**) cloudy conditions represent migration toward nests, and (**c**) rainy conditions represent short-range foraging around nests. A Markov controller translates these three abstractions into recurrent search modes.

Figure 1 makes this separation explicit. The observations motivate three regimes, whereas Section 3.2 and Section 3.3 provide their independent mathematical definitions.

### 3.2. Weather State Machine: Markov Process

Consider a finite state space $S=\{s_{1},s_{2},\ldots ,s_{n}\}$. The variables $X_{t}$ and $X_{0}$ denote the states at generation *t* and initialization, respectively. For any candidate state $s_{j}\in S$, first-order dependence requires

(1) $$P\left(X_{t+1}=s_{j}|X_{t},X_{t-1},\ldots ,X_{0}\right)=P\left(X_{t+1}=s_{j}|X_{t}\right).$$

The entry $p_{ij}$ of the one-step matrix $P={\left[p_{ij}\right]}_{n\times n}$ specifies a transition from $s_{i}$ to $s_{j}$. Each row is a probability distribution:

(2) $$p_{ij}=P\left(X_{t+1}=s_{j}|X_{t}=s_{i}\right),\;\sum_{j=1}^{n}p_{ij}=1,\;p_{ij}\geq 0.$$

WSAO uses $S=\{s_{1}=\mathrm{sunny},s_{2}=\mathrm{cloudy},s_{3}=\mathrm{rainy}\}$ and the following numerical transition matrix:

(3) $$P=\left[\begin{array}{ccc} 0.6 & 0.25 & 0.15\\ 0.25 & 0.25 & 0.50\\ 0.25 & 0.05 & 0.70 \end{array}\right]$$

Both matrix dimensions follow the order $\left(s_{1},s_{2},s_{3}\right)=\left(\mathrm{sunny},\mathrm{cloudy},\mathrm{rainy}\right)$.

**Figure 2:**
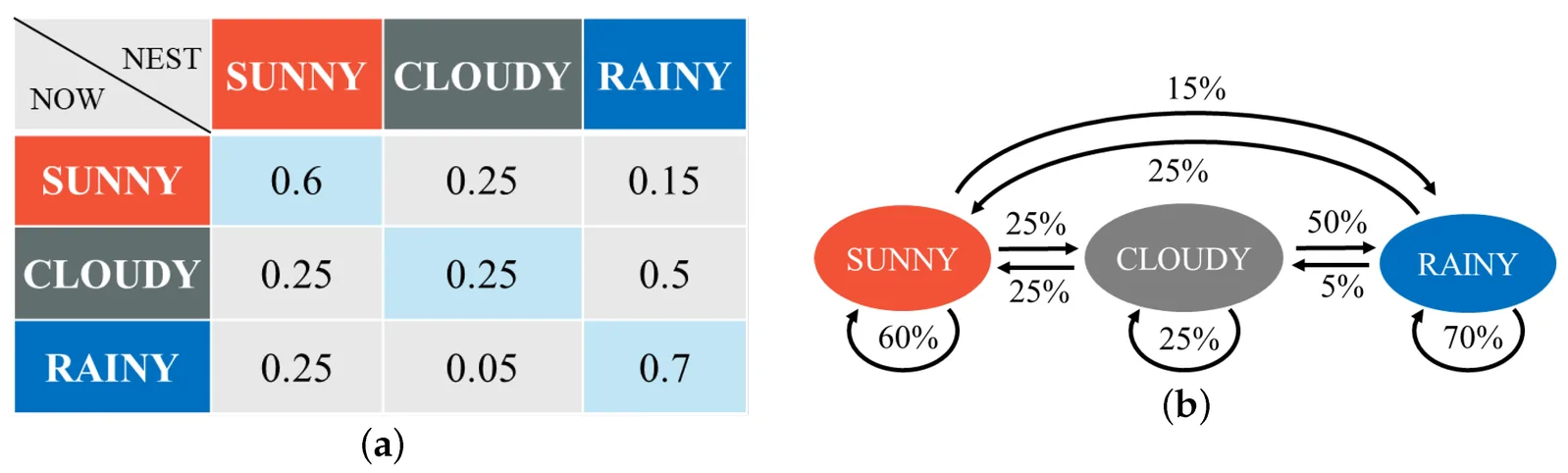
Weather-state controller used by WSAO. (**a**) Transition matrix P. Red, gray, and blue headers denote sunny, cloudy, and rainy states, respectively; light-blue diagonal cells denote self-transition probabilities, whereas light-gray off-diagonal cells denote transitions between different states. (**b**) Directed transition graph for the three modes; each edge is weighted by the corresponding transition probability.

Figure 2 presents the same controller as a matrix and as a directed transition graph.

The probabilities define a reproducible baseline, not a theoretically optimal matrix. Self-transition values of 0.60 for sunny and 0.70 for rainy sustain short episodes of exploration and refinement. The 0.50 cloudy-to-rainy entry favors refinement after nest congregation. A 0.25 return from either local state to sunny preserves recurrent global search. Every run starts from sunny, $X_{0}=s_{1}$. Using one fixed matrix across benchmarks isolates state switching from any additional feedback adaptation.

All entries of P are positive, so the chain is irreducible and aperiodic and admits one stationary distribution. Solving πP=π yields π=(0.3846,0.1587,0.4567) for sunny, cloudy, and rainy. The expected episode lengths, $1/(1-p_{ii})$, are 2.50, 1.33, and 3.33 generations. From either cloudy or rainy, the mean first passage to sunny is four transitions. Section 4.5 tests whether alternative dynamics materially change optimization on selected functions.

### 3.3. Nest Systems and Agent Behavior

#### 3.3.1. Initialization

Let *N* denote population size and $x_{i}^{\left(0\right)}$ the starting position of agent *i*. Component-wise bounds are $\mathrm{LB},\mathrm{UB}\in R^{D}$. With independent $U_{i}^{\left(0\right)}\in {\left[0,1\right]}^{D}$ and element-wise multiplication ⊙, initialization is

(4) $$x_{i}^{\left(0\right)}=\mathrm{LB}+U_{i}^{\left(0\right)}⊙\left(\mathrm{UB}-\mathrm{LB}\right),\;i=1,\ldots ,N$$

Every initial velocity is zero, $v_{i}^{\left(0\right)}=0$. Each component is subsequently limited to [−0.1(UB−LB),0.1(UB−LB)]. An infeasible position component is clipped to its nearest bound. Clipping neither resets nor reflects velocity. Cognitive, social, and nest terms can redirect the next update, while boundary optima remain reachable.

#### 3.3.2. Nest Determination

Nest diversity is controlled by a minimum separation $r_{\min}$. For an *n*-dimensional ball of radius *r*, volume $V_{n}\left(r\right)$ is expressed using the gamma function Γ(·):

(5) $$V_{n}\left(r\right)=\frac{\pi^{n/2}}{\Gamma (n/2+1)}\cdot r^{n}$$

Here, k>0 is a dimensionless nominal coverage coefficient, *D* is the dimension, and $n_{\mathrm{Nest}}$ is the requested nest count. Bounds in coordinate *d* are ${\mathrm{LB}}_{d}$ and ${\mathrm{UB}}_{d}$. Let $V_{\mathrm{domain}}=\prod_{d=1}^{D}\left({\mathrm{UB}}_{d}-{\mathrm{LB}}_{d}\right)$. We define *k* through the aggregate nominal-volume relation $n_{\mathrm{Nest}}V_{D}\left(r_{\min}\right)=kV_{\mathrm{domain}}$. Solving this relation for $r_{\min}$ gives

(6) $$r_{\min}={\left(\frac{k\cdot \prod_{d=1}^{D}\left({\mathrm{UB}}_{d}-{\mathrm{LB}}_{d}\right)}{n_{\mathrm{Nest}}\cdot V_{D}\left(1\right)}\right)}^{1/D}$$

The term $V_{D}\left(1\right)$ is the *D*-dimensional unit-ball volume. Thus, increasing *k* increases the separation scale. It is not an exact packing fraction because boundary truncation and fallback filling can relax the geometric construction. The value k=0.4 was selected as a cross-function compromise in the sensitivity analysis of Section 4.4; it is not presented as a theoretical optimum. At generation *t*, archive $C^{\left(t\right)}$ stores evaluated candidates in ascending fitness order. Algorithm 1 accepts a screened candidate only if its decision-space distance to every existing nest is at least $r_{\min}$.
**Algorithm 1** Distance-screened nest selection.**Input:** Fitness-ordered archive C, separation threshold $r_{\min}$, and requested nest count $n_{\mathrm{Nest}}$ **Output:** Selected centers S
Initialize the center set as S←⌀.**for each** $c_{i}$ in the ordered archive C **do**    Accept $c_{i}$ when $∥c_{i}{-s∥}_{2}\geq r_{\min}$ for every s∈S.    On acceptance, set $S\leftarrow S\cup \{c_{i}\}$.    Stop the scan once $\left|S\right|=n_{\mathrm{Nest}}$.**end for**If $|S|<n_{\mathrm{Nest}}$, draw archived candidates to fill the remaining entries.**return** the completed set S.


If strict screening returns too few centers, the final safeguard fills the missing entries by resampling archived candidates. This branch relaxes the separation rule. Pairwise distance is therefore guaranteed only for nests admitted during primary screening.

#### 3.3.3. Sunny State: Global Exploration

Sunny weather applies a PSO-inspired exploratory update with generation-dependent weights. For agent *i*, independent scalars $\rho_{1i}^{\left(t\right)},\rho_{2i}^{\left(t\right)}∼U\left(0,1\right)$ are broadcast across all *D* coordinates:

(7) $$\begin{array}{cc} v_{i}^{\left(t+1\right)} & =w^{\left(t\right)}v_{i}^{\left(t\right)}+c_{1}\rho_{1i}^{\left(t\right)}\left({\mathrm{pbest}}_{i}^{\left(t\right)}-x_{i}^{\left(t\right)}\right)+c_{2}^{\left(t\right)}\rho_{2i}^{\left(t\right)}\left({\mathrm{gbest}}^{\left(t\right)}-x_{i}^{\left(t\right)}\right)\\ x_{i}^{\left(t+1\right)} & =x_{i}^{\left(t\right)}+v_{i}^{\left(t+1\right)} \end{array}$$

The vectors $v_{i}^{\left(t\right)}$ and $x_{i}^{\left(t\right)}$ denote velocity and position. Personal and global bests are ${\mathrm{pbest}}_{i}^{\left(t\right)}$ and ${\mathrm{gbest}}^{\left(t\right)}$. The settings are $c_{1}=1.6$, $w^{\left(t\right)}=0.9-0.8t/T$, and $c_{2}^{\left(t\right)}=0.4+1.2t/T$.

The PSO-derived form is deliberate [16]. Velocity memory and the personal and global records provide a stable population-wide information channel for the sunny state, complementing the nest-directed cloudy and rainy updates. The contribution of WSAO therefore lies in recurrent Markov switching and the integration of complementary operators, rather than in presenting the PSO velocity law itself as new.

#### 3.3.4. Cloudy State: Nest Congregation

Once the first nest set is available, every agent is assigned one nest uniformly at random. Assignments are redrawn during each cloudy generation:

(8) $${\mathrm{nest}}_{i}^{\left(t\right)}=S\left[\;\mathrm{randi}\left(\left[1,n_{\mathrm{Nest}}\right]\right)\;\right]$$

The set S contains current nests, and S[q] is its *q*-th member. The operator $\mathrm{randi}(\left[1,n_{\mathrm{Nest}}\right])$ samples a uniform integer. Rainy weather reuses the latest assignment, including when the first state change goes directly from sunny to rainy. Cloudy updates combine the assigned nest with the personal best. Independent vectors $r_{1i}^{\left(t\right)},r_{3i}^{\left(t\right)}∼U\left({\left[0,1\right]}^{D}\right)$ weight these directions:

(9) $$\begin{array}{cc} v_{i}^{\left(t+1\right)} & =w^{\left(t\right)}v_{i}^{\left(t\right)}+c_{1c}r_{1i}^{\left(t\right)}⊙\left({\mathrm{pbest}}_{i}^{\left(t\right)}-x_{i}^{\left(t\right)}\right)+c_{3c}r_{3i}^{\left(t\right)}⊙\left({\mathrm{nest}}_{i}^{\left(t\right)}-x_{i}^{\left(t\right)}\right)\\ x_{i}^{\left(t+1\right)} & =x_{i}^{\left(t\right)}+v_{i}^{\left(t+1\right)} \end{array}$$

Agent *i* follows ${\mathrm{nest}}_{i}^{\left(t\right)}$ with $c_{3c}=2.0$ and retains personal-best attraction with $c_{1c}=0.6$. The inertia $w^{\left(t\right)}=0.9-0.8t/T$ matches the sunny schedule.

#### 3.3.5. Rainy State: Local Exploitation

Rainy weather preserves personal-best and nest guidance but scales the realized displacement. Let $\eta_{r}$ denote this position factor. Independent vectors $r_{1i}^{\left(t\right)},r_{3i}^{\left(t\right)}∼U\left({\left[0,1\right]}^{D}\right)$ weight the two attractors:

(10) $$\begin{array}{cc} v_{i}^{\left(t+1\right)} & =w^{\left(t\right)}v_{i}^{\left(t\right)}+c_{1r}r_{1i}^{\left(t\right)}⊙\left({\mathrm{pbest}}_{i}^{\left(t\right)}-x_{i}^{\left(t\right)}\right)+c_{3r}r_{3i}^{\left(t\right)}⊙\left({\mathrm{nest}}_{i}^{\left(t\right)}-x_{i}^{\left(t\right)}\right)\\ x_{i}^{\left(t+1\right)} & =x_{i}^{\left(t\right)}+\eta_{r}v_{i}^{\left(t+1\right)} \end{array}$$

The implemented values are $c_{1r}=c_{3r}=1.6$, $w^{\left(t\right)}=0.9-0.8t/T$, and $\eta_{r}=0.5$. Rainy weather therefore uses the cloudy attractor types with stronger personal guidance and half displacement. The global best is absent from this equation. Section 4.5 compares the implemented factor with $\eta_{r}=1.0$.

#### 3.3.6. Global/Differential Trial Perturbation

After the weather update is evaluated, ${\tilde{\mathrm{pbest}}}_{i}^{\left(t+1\right)}$ denotes the refreshed personal best. WSAO then proposes one of two trial moves. The global branch samples a domain-wide displacement. The differential branch uses two updated personal-best vectors:

(11) $$x_{i,\mathrm{try}}^{\left(t+1\right)}=\left\{\begin{array}{cc} {\tilde{\mathrm{pbest}}}_{i}^{\left(t+1\right)}+c_{\ell 1}^{\left(t\right)}\left[x_{\min}+r_{1}⊙\left(x_{\max}-x_{\min}\right)\right], & \mathrm{global}\;\mathrm{trial},\\ {\tilde{\mathrm{pbest}}}_{i}^{\left(t+1\right)}+\left[c_{\ell 2}\left(1-\rho \right)+\rho \right]\left({\tilde{\mathrm{pbest}}}_{r_{3}}^{\left(t+1\right)}-{\tilde{\mathrm{pbest}}}_{r_{4}}^{\left(t+1\right)}\right), & \mathrm{differential}\;\mathrm{trial}. \end{array}\right.$$

The proposed vector is $x_{i,\mathrm{try}}^{\left(t+1\right)}$, and $x_{\min},x_{\max}$ are the bounds. Random quantities satisfy $r_{1}∼U\left({\left[0,1\right]}^{D}\right)$ and ρ∼U(0,1). Indices $r_{3},r_{4}$ are obtained from independent permutations of personal bests. The coefficient $c_{\ell 1}^{\left(t\right)}={\left(1-t/T\right)}^{2t/T}$ approaches zero as the run ends. With $c_{\ell 2}=0.2$, the differential multiplier $c_{\ell 2}\left(1-\rho \right)+\rho$ remains in [0.2,1]. The global branch has probability 0.2; all other trials use the differential branch. Each trial is clipped, evaluated, and compared with the refreshed personal best:

(12) $$\left(x_{i}^{\left(t+1\right)},{\mathrm{pbest}}_{i}^{\left(t+1\right)}\right)\leftarrow \left\{\begin{array}{cc} \left(x_{i,\mathrm{try}}^{\left(t+1\right)},x_{i,\mathrm{try}}^{\left(t+1\right)}\right), & f\;\left(x_{i,\mathrm{try}}^{\left(t+1\right)}\right)<f\;\left({\tilde{\mathrm{pbest}}}_{i}^{\left(t+1\right)}\right),\\ \left(x_{i}^{\left(t+1\right)},{\tilde{\mathrm{pbest}}}_{i}^{\left(t+1\right)}\right), & \mathrm{otherwise}. \end{array}\right.$$

This acceptance rule adds long-range and differential proposals while protecting improvements already obtained from the active weather equation.

### 3.4. Complete WSAO Algorithm

Algorithm 2 summarizes the complete WSAO procedure.
**Algorithm 2** Weather State Ants Optimizer.**Input:** Agent count *N*, generation limit *T*, search bounds LB and UB, dimension *D*, and objective *f* **Output:** Incumbent decision vector $x^{*}$ with value $f(x^{*})$
Draw the initial population from Equation (4); obtain $r_{\min}$ from Equation (6).Evaluate the population and establish every personal best and the initial global best.Start the controller in the SUNNY mode.**for** generation t=1,…,T **do**    Build the current center set with Algorithm 1.    Create nest assignments after centers first become available; redraw them from Equation (8) in each CLOUDY generation.    Execute Equation (7), (9), or (10), according to the active mode.    Draw the subsequent mode using the active row of P.    Enforce the bounds, evaluate the weather update, and refresh personal bests.    Generate the trial in Equation (11) and apply the acceptance rule in Equation (12).    Refresh the global incumbent.**end for****return** $x^{*}$ and $f(x^{*})$.


**Figure 3:**
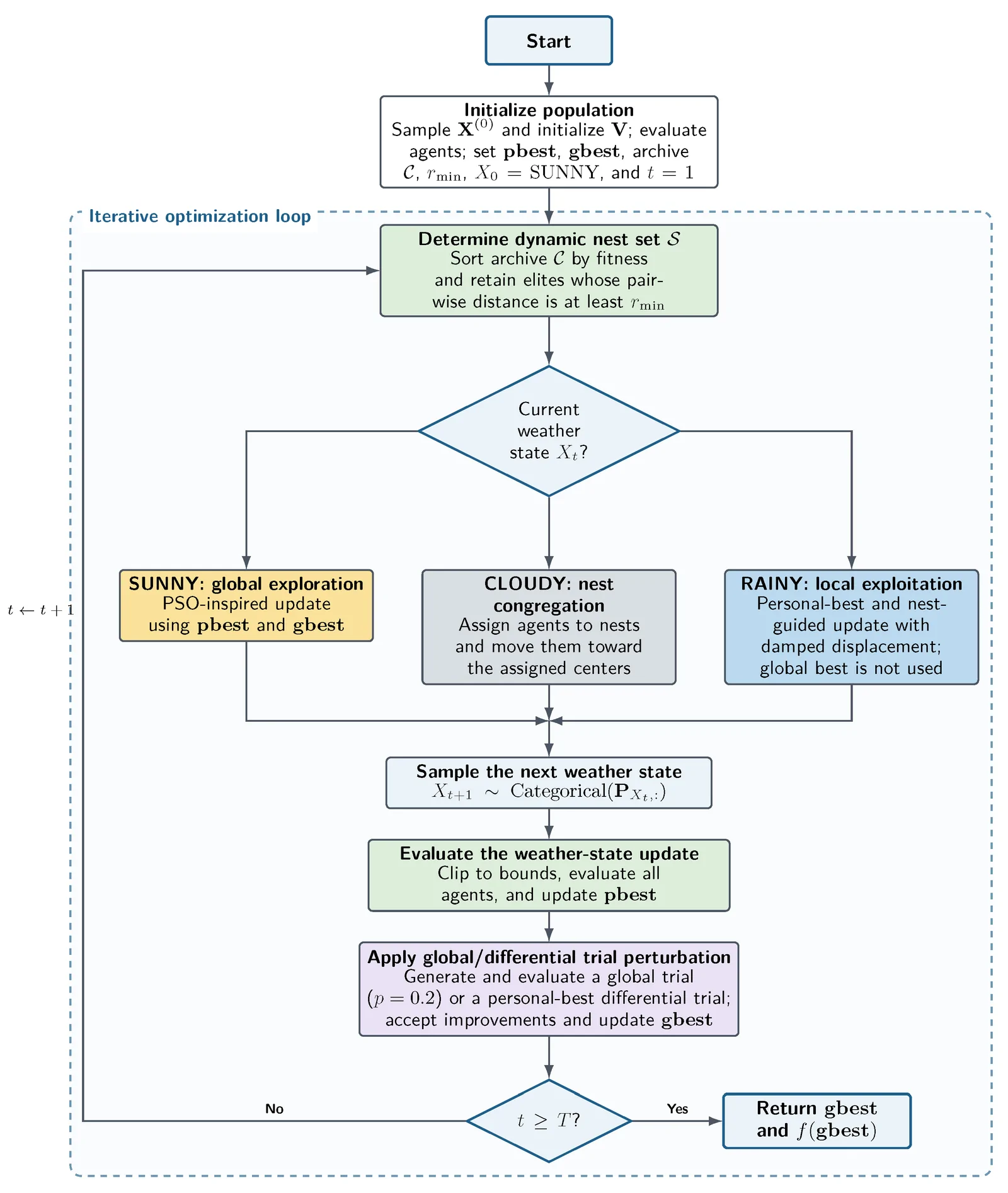
One WSAO generation from state selection to incumbent update. Yellow, gray, and blue boxes denote sunny, cloudy, and rainy updates; green boxes denote archive or evaluation operations; purple denotes the trial perturbation; pale blue denotes control and termination decisions; and white denotes common initialization. After sampling the following mode, WSAO evaluates the bounded weather update, refreshes personal records, tests a global or differential proposal, and finally updates the global incumbent.

**Figure 4:**
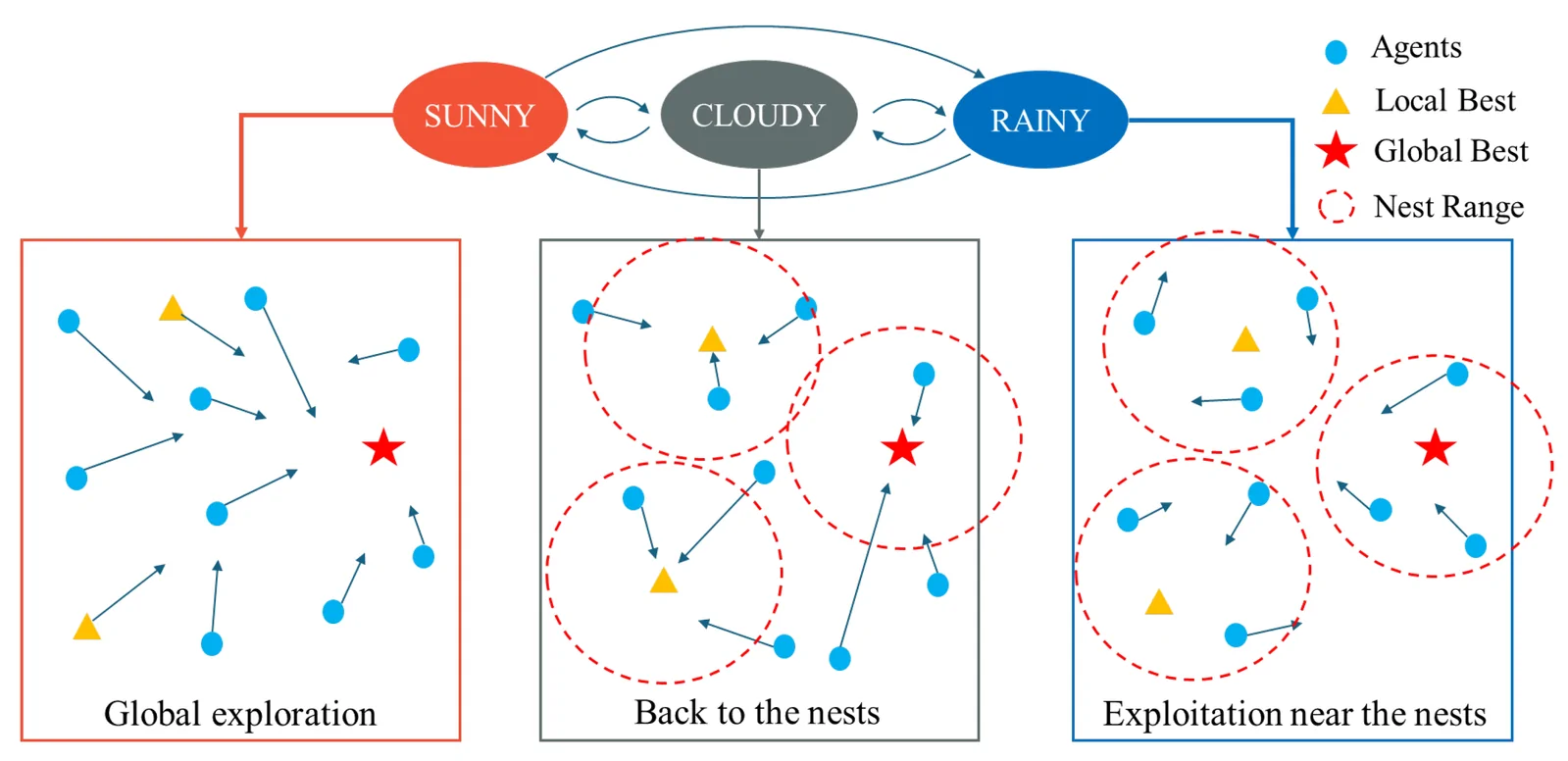
Operational structure of WSAO. Curved bidirectional arrows denote recurrent Markov transitions among the weather states. Downward arrows connect each state to its population update, and arrows inside the lower panels show agent motion toward the global best, personal bests, or assigned nests. The multi-nest archive supplies several spatially separated elite centers for parallel regional refinement.

Figure 3 follows one complete generation. Initialization sets the population and sunny state. The loop screens historical candidates for nests and applies the equation selected by current weather. The controller then samples the following state from the corresponding row of P. After clipping and objective evaluation, personal bests are refreshed. Trial candidates are evaluated against these values before the global best is updated. Figure 4 provides a complementary structural view of the three modes.

Figure 4 shows the component interfaces. The state machine chooses one population equation. The archive supplies nests to cloudy and rainy updates, and the trial operator follows every weather-specific update.

Table 2 collects every fixed or scheduled WSAO setting. One configuration was used throughout both CEC suites, with no function-specific tuning.

### 3.5. Computational Complexity

Let $C_{f}$ be the cost of one objective evaluation and $A_{t}$ the archive size at generation *t*. Objective evaluation and state equations require $O\;(NC_{f}+ND)$ per generation. Updating the ordered archive requires $O\;(N\log N+A_{t})$, while worst-case nest screening requires $O\;(A_{t}n_{\mathrm{Nest}}D)$. The total time is $O\;\left(\sum_{t=1}^{T}\left[NC_{f}+ND+N\log N+A_{t}+A_{t}n_{\mathrm{Nest}}D\right]\right)$. The unpruned implementation has $A_{t}=tN$, so its time and archive memory are $O\;(TNC_{f}+TN\log N+NT^{2}n_{\mathrm{Nest}}D)$ and O(NTD). The quadratic term matters when *T* becomes large. Our experiments use T=500 and $n_{\mathrm{Nest}}=5$. Incremental hashing can remove exact *D*-dimensional duplicates in expected O(ND) time per generation. However, deduplication cannot bound a continuous archive whose points are mostly distinct. Capping the deduplicated archive at *B* elite or diversity-preserving candidates changes maintenance to O((B+N)logB) and nest screening to $O\;(Bn_{\mathrm{Nest}}D)$ per generation. The expected total time then becomes

$$O\;\left(T[NC_{f}+ND+N\log N+\left(B+N\right)\log B+Bn_{\mathrm{Nest}}D]\right),$$

with O(BD) archive memory. For fixed *B*, the dependence on *T* is linear. This bounded design is an implementation extension and did not generate the reported results.

## 4. Experimental Results and Analysis

### 4.1. Experimental Setup

The benchmark programs ran in MATLAB R2022b under 64-bit Windows 11 on an AMD Ryzen 7 5800H at 3.20 GHz with 16 GB RAM. Every run used N=100 agents and T=500 generations, corresponding to a nominal MaxFEs=50,000. Each function-algorithm pair was repeated independently 30 times.

The numerical study covered two established suites:

- **IEEE CEC2017**: F1 and F3–F30, giving 29 functions after the standard exclusion of F2. The set contains unimodal, multimodal, hybrid, and composition categories.
- **IEEE CEC2022**: all 12 functions, comprising one unimodal function, four multimodal functions, three hybrids, and four compositions.

All 41 functions were evaluated at D=10 to keep dimensionality identical across suites and algorithms.

### 4.2. Comparison Algorithms

The comparison set comprised 11 algorithms spanning four groups:

- **Classic**: PSO [16], GWO [45], WOA [48], MPA [8]
- **Recent metaheuristics**: Artificial Hummingbird Algorithm (AHA) [52], Kepler Optimization Algorithm (KOA) [27], Superb Fairy-wren Optimization Algorithm (SFOA) [53], and Enzyme Action Optimizer (EAO) [54]
- **Differential evolution variants**: LSHADE [33], LSHADE-EpSin [35]
- **Enhanced variant**: Velocity Pausing Particle Swarm Optimization (VPPSO) [47].

Population size, generation count, and nominal evaluation budget were matched for all methods. Comparator settings followed their cited implementations and appear in Table 3. Table 2 reports the WSAO settings.

Table 3 makes the comparator configuration auditable. Each listed setting remained fixed across functions, and the common run limits matched the WSAO protocol.

### 4.3. Performance Metrics

Performance was summarized at run, function, and suite levels:

- **Average (Avg)** and **standard deviation (Std)** describe the final best fitness across 30 runs.
- **Friedman mean rank** orders algorithms across a complete suite; a smaller rank is better.
- **Two-sided Wilcoxon rank-sum tests** compare WSAO with each competitor on each function, using family-wise α=0.05.

CEC2017 and CEC2022 formed two predeclared multiplicity families. Holm’s step-down method jointly adjusted every WSAO-versus-competitor contrast within each suite. The tables report $p_{\mathrm{adj}}$ and bold values below 0.05. Interpretation combines these tests with Avg, Std, and the Friedman ordering.

### 4.4. Parameter Sensitivity Analysis

**Figure 5:**
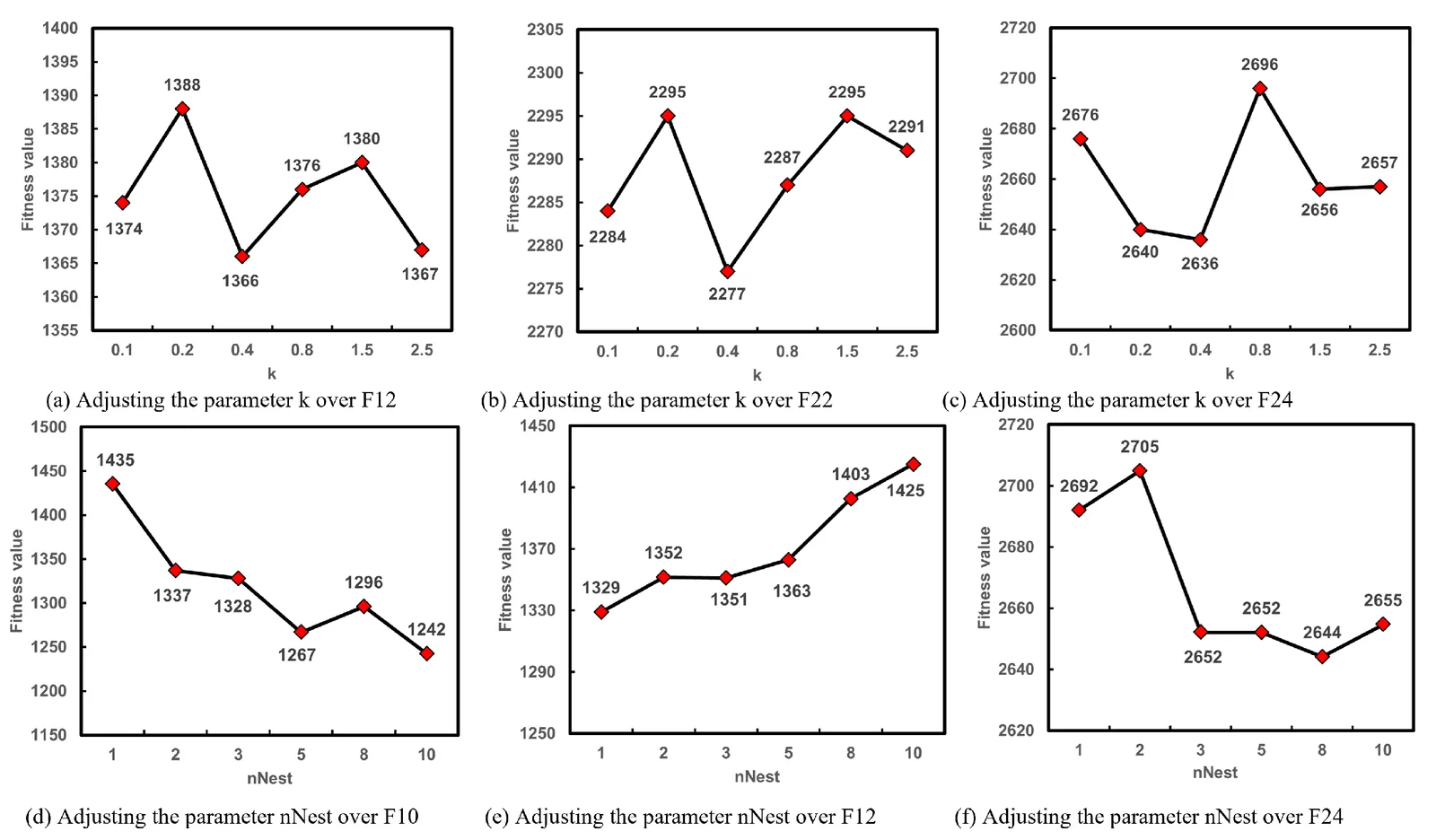
Response of WSAO to its nest parameters. Panels (**a**–**c**) vary the coverage coefficient *k* on F12, F22, and F24; panels (**d**–**f**) vary the requested count $n_{\mathrm{Nest}}$ on F10, F12, and F24.

A preliminary study varied nest coverage *k* and nest count $n_{\mathrm{Nest}}$ at D=10 on CEC2017 F10, F12, F22, and F24. Tested coverage values were {0.1,0.2,0.4,0.8,1.5,2.5}, and nest counts were {1,2,3,5,8,10}. Figure 5 identifies k=0.4 and $n_{\mathrm{Nest}}=5$ as balanced settings across these functions. The remaining experiments used this pair.

Figure 5a–c show function-dependent sensitivity to *k*. On F12 and F22, k=0.4 gives the lowest or near-lowest final values among the tested settings, while on F24 it avoids the pronounced deterioration observed at k=0.8. Figure 5d–f further show that the effect of the requested nest count depends on landscape structure: F10 improves as the count increases from one, F12 favors a single center and deteriorates at larger counts, and F24 performs poorly with one or two centers but improves once several centers are available. Five nests therefore serve as a cross-function compromise that provides moderate multi-center capacity without claiming to be individually optimal for every function.

### 4.5. Targeted Markov Sensitivity and Component Ablation

Targeted controls assessed the matrix, temporal dependence, nest count, trial operator, and rainy displacement. CEC2022 F1, F4, F7, F10, and F12 represent unimodal, multimodal, hybrid, and composition cases at D=10. Every configuration used N=100, T=500, and the same 30 function-specific seeds. Final error above the known optimum was the primary endpoint. Two-sided Wilcoxon signed-rank tests analyzed paired $\log_{10}\left(\mathrm{error}+10^{-12}\right)$ differences. Holm correction was applied separately to the matrix, component, and rainy-factor families.

Three alternatives were compared with P. Low persistence used (P−0.15I)/0.85, while high persistence used 0.25I+0.75P. Both changed episode duration without changing the stationary distribution. Strong recovery replaced the rainy row by (0.45,0.05,0.50). Further controls sampled states independently from the baseline stationary distribution, retained one nest, removed the trial displacement under a matched budget, or set $\eta_{r}=1.0$.

Full WSAO occupied the sunny, cloudy, and rainy states for empirical fractions of 0.3884, 0.1604, and 0.4513, respectively. Mean episode lengths were 2.521, 1.336, and 3.343 generations, consistent with matrix expectations. Detectable matrix effects occurred only on F1. Low persistence raised the median paired log-error by 0.694, whereas high persistence lowered it by 0.738. Neither variant differed on F4, F7, F10, or F12 after correction. Strong recovery differed on none of the five functions. No tested matrix therefore improved the full selected set.

Independent state sampling increased F1 error, with no detected difference on the remaining functions. One nest improved F1, did not change F10 detectably, and worsened F4, F7, and F12. Multiple centers therefore benefited the structured cases in this subset, but not the unimodal case. Removing the trial displacement under a matched budget increased every function’s error and lost 136 of 150 paired runs.

Removing rainy damping by setting $\eta_{r}=1.0$ increased F1 error and caused no adjusted difference elsewhere. The undamped control recorded 57 wins, 90 losses, and three ties over 150 pairs. We therefore retain 0.5 as a conservative displacement factor without claiming universal optimality.

Table 4 consolidates the matrix-sensitivity and component-ablation results.

### 4.6. Convergence Behavior Analysis

**Figure 6:**
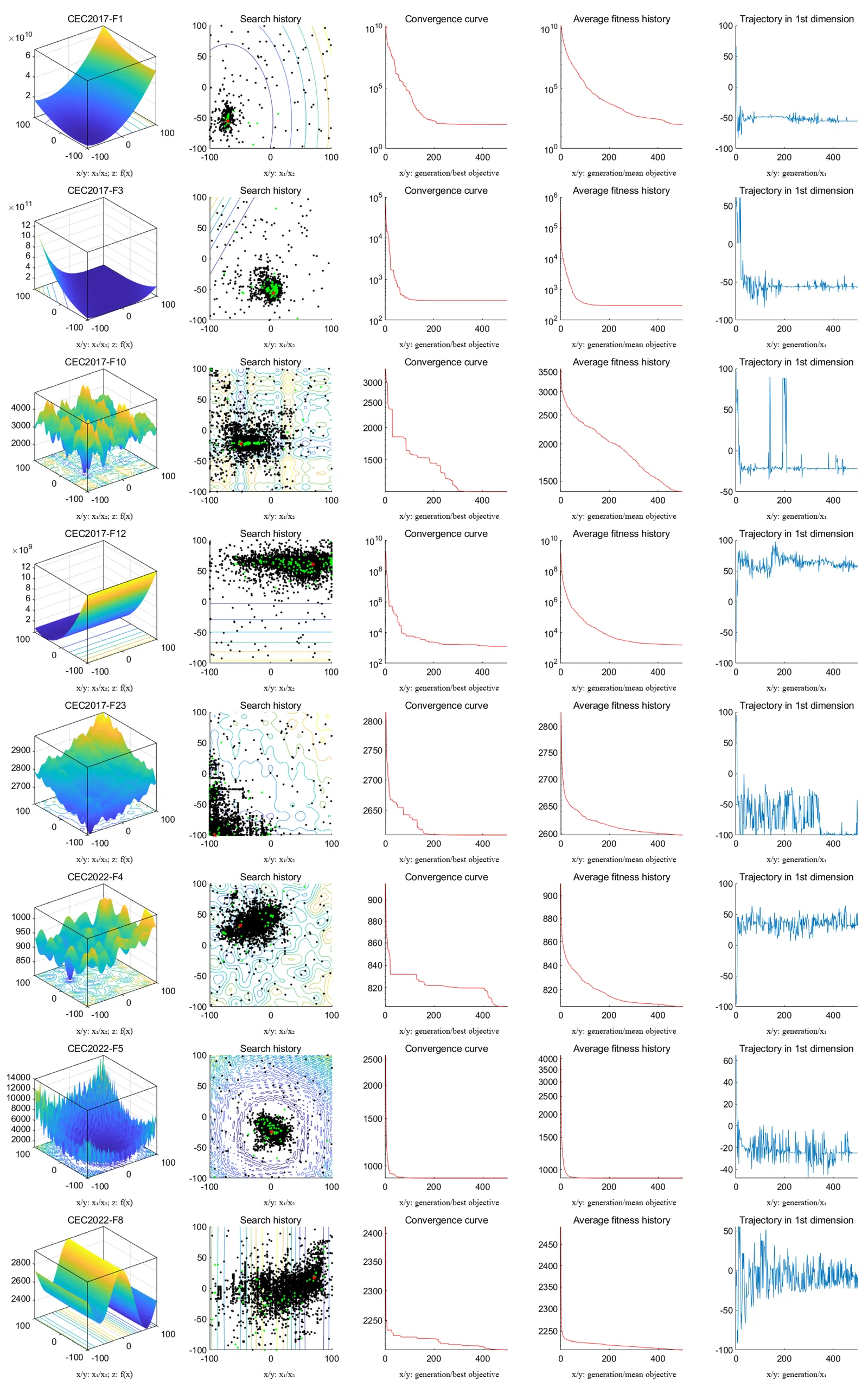
Representative WSAO runs at D=10 for CEC2017 F1, F3, F10, F12, and F23 and CEC2022 F4, F5, and F8. From left to right, each row presents the landscape, sampled locations, incumbent-value history, population-mean history, and agent paths. The axis keys beneath each panel give the horizontal/vertical quantities; the landscape panels additionally use f(x) on the vertical axis. Decision variables and CEC objective values are dimensionless, and history panels use generation on the horizontal axis.

Figure 6 combines five views from representative D=10 runs: landscape, search history, best fitness, population-average fitness, and one agent trajectory. These panels show domain coverage, retained regions, and progressive refinement. They complement the 30-run summaries in Section 4.7 and Section 4.8.

The first panel in each row displays the first two coordinates, with all others fixed at the plotted run’s best solution. Search-history panels overlay agents and nests to reveal whether several candidate regions persist. Best and average fitness appear in the third and fourth panels. Smooth declines indicate gradual refinement, while steps mark abrupt improvements between candidate basins. The final panel traces one agent’s first coordinate. Its repeated jumps are consistent with nest reassignment and trial perturbations. Together, the views show alternating broad movement and localized refinement.

### 4.7. CEC2017 Benchmark Results

Table 5 reports the D=10 CEC2017 ordering. WSAO obtained mean rank 2.48, followed by LSHADE-EpSin at 2.50, MPA at 2.79, and EAO at 3.12. WSAO placed first on eight functions and within the top three on 25 of 29. Table 6 gives every Avg, Std, and function rank. Table 7 gives the adjusted pairwise tests. Of 319 contrasts, 278 remained significant after Holm correction. Directional classification yielded 248 wins, 41 ties, and 30 losses for WSAO.

### 4.8. CEC2022 Benchmark Results

The CEC2022 summary in Table 8 gives WSAO the lowest mean rank, 2.33. EAO and LSHADE-EpSin follow at 2.54 and 2.71. WSAO led five functions and ranked within the top three on 10 of 12. Complete descriptive results appear in Table 9. Adjusted contrasts appear in Table 10. Holm correction retained significance for 119 of 132 tests. The resulting WSAO counts were 103 wins, 13 ties, and 16 losses.

### 4.9. Convergence Speed Comparison

**Figure 7:**
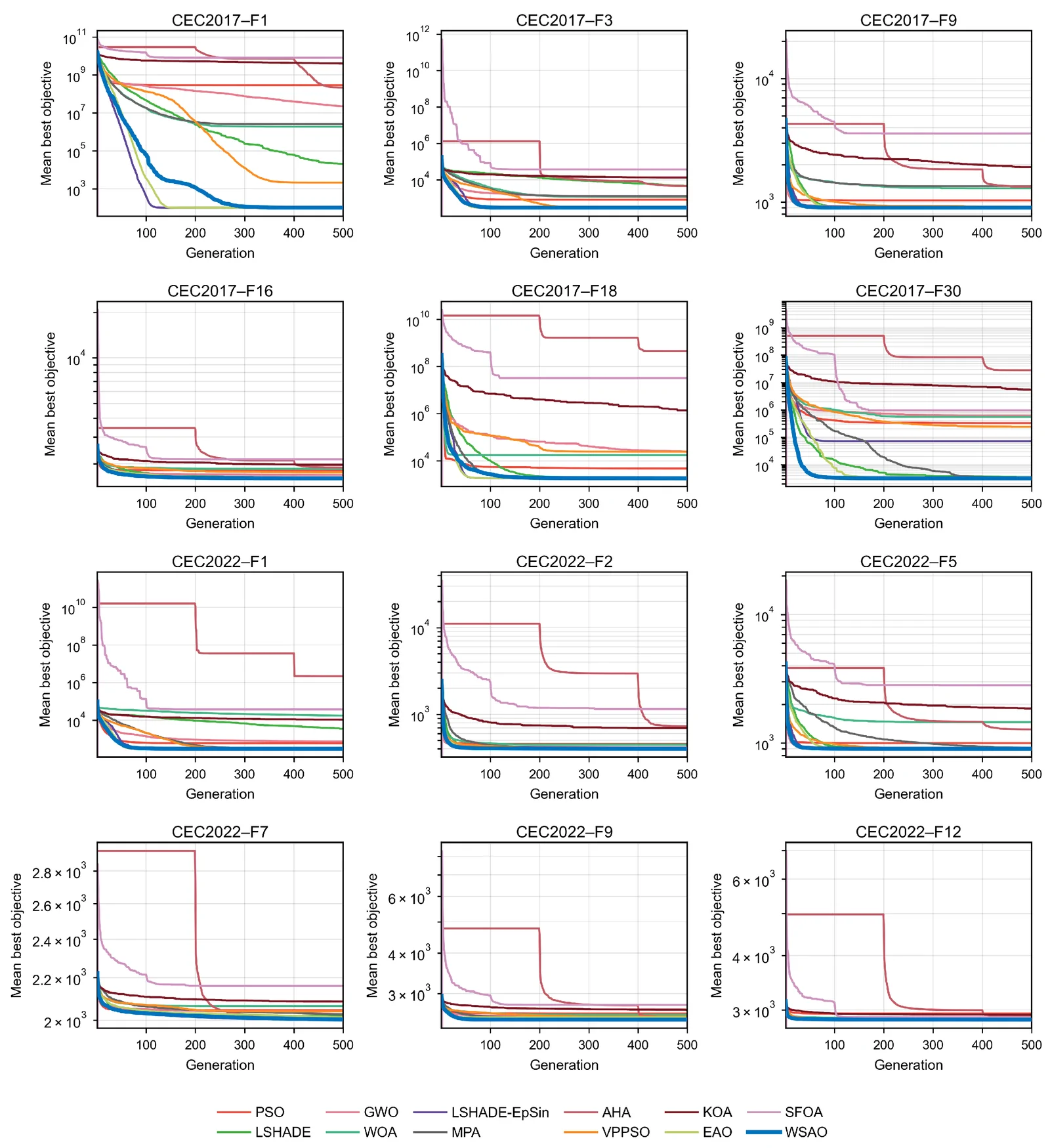
Mean convergence histories from 30 independent D=10 runs. The selected cases are CEC2017 F1, F3, F9, F16, F18, and F30 and CEC2022 F1, F2, F5, F7, F9, and F12. The horizontal axis is generation and the vertical axis is the dimensionless mean best-so-far CEC objective value.

Figure 7 compares 30-run average best-so-far curves for all 12 algorithms. Selected panels span unimodal, multimodal, hybrid, and composition functions from both suites. Unlike the terminal ranks, these traces reveal when improvements occurred under the common budget and whether an early lead persisted.

## 5. Engineering Applications

Five constrained benchmarks extend the evaluation to the welded beam, tension/compression spring, pressure vessel, three-bar truss, and cantilever beam design problems. Their nonlinear objectives, dimensions, and inequality structures differ. The shared protocol used 30 runs, N=100, T=500, and a nominal limit of 50,000 evaluations for every algorithm.

Each case has the following constrained single-objective form:

(13) $$\min_{x\in \Omega }f\left(x\right)\;\mathrm{subject}\;\mathrm{to}\;g_{j}\left(x\right)\leq 0,\;j=1,\ldots ,m,$$

Here, x is the design vector, Ω is its bounded domain, f(x) is the objective, and $g_{j}\left(x\right)$ is constraint *j*. Infeasible candidates receive a static exterior penalty:

(14) $$F\left(x\right)=f\left(x\right)+\lambda \sum_{j=1}^{m}\max{\left\{0,g_{j}\left(x\right)\right\}}^{q},$$

The penalized fitness is F(x), with coefficient λ and exponent *q*. Welded-beam design used $\left(\lambda ,q\right)=\left(10^{5},2\right)$, and the spring used $\left(10^{2},2\right)$. The other three cases used $\left(10^{5},1\right)$. For a given problem, WSAO and all comparators received the identical function handle, including the same objective, constraints, coefficient, and exponent. Constraint handling was therefore algorithm-independent. The unconstrained CEC suites did not use Equation (14). Each following subsection specifies the formulation and results. Best denotes the lowest feasible objective from 30 runs. Avg and Std summarize all final objectives, and Rank follows Avg. Tabulated variables are rounded, so recomputation from displayed values can differ slightly from the recorded objective.

### 5.1. Welded Beam Design

**Figure 8:**
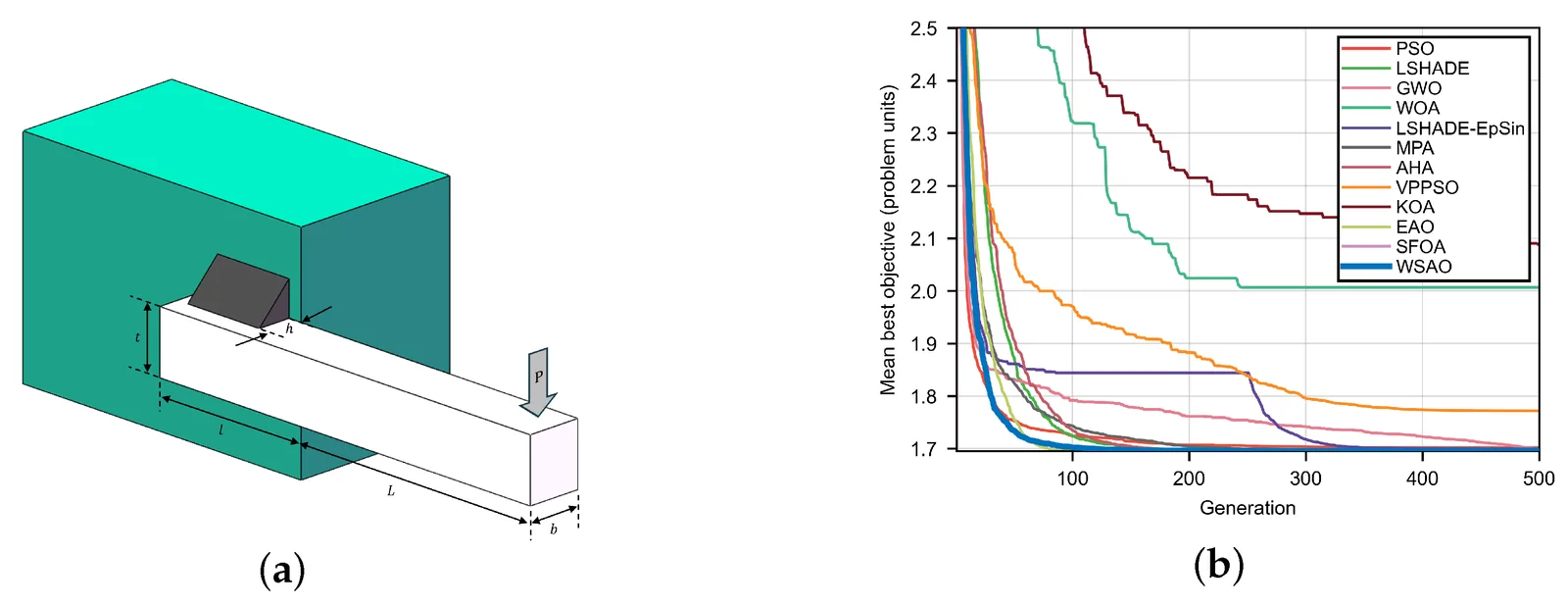
Welded-beam case: (**a**) geometry and decision variables; (**b**) mean convergence histories across 30 runs. In panel (**b**), the horizontal axis is generation and the vertical axis is the mean best objective value in the problem-specific units defined above.

Figure 8a shows the welded-beam benchmark. Manufacturing cost is minimized under shear-stress τ, bending-stress σ, buckling-load $P_{c}$, deflection δ, and geometric constraints. The vector $x=\left[x_{1},x_{2},x_{3},x_{4}\right]=\left[h,l,t,b\right]$ contains weld thickness, weld length, beam height, and beam width. Following [21], the objective is

(15) $$\min f\left(x\right)=1.10471x_{1}^{2}x_{2}+0.04811x_{3}x_{4}\left(14+x_{2}\right),$$

where f(x) is manufacturing cost. The inequalities are

(16) $$\begin{array}{cc} g_{1}\left(x\right)=\tau \left(x\right)-\tau_{\max}\leq 0, & g_{2}\left(x\right)=\sigma \left(x\right)-\sigma_{\max}\leq 0,\\ g_{3}\left(x\right)=\delta \left(x\right)-\delta_{\max}\leq 0, & g_{4}\left(x\right)=x_{1}-x_{4}\leq 0,\\ g_{5}\left(x\right)=P-P_{c}\left(x\right)\leq 0, & g_{6}\left(x\right)=0.125-x_{1}\leq 0,\\ g_{7}\left(x\right)=1.10471x_{1}^{2}x_{2}+0.04811x_{3}x_{4}\left(14+x_{2}\right)-5\leq 0. & \end{array}$$

The auxiliary stress, deflection, and buckling quantities are

(17) $$\begin{array}{cccc} \tau (x) & =\sqrt{{\left(\tau^{\prime }\right)}^{2}+2\tau^{\prime }\tau^{\prime \prime }\frac{x_{2}}{2R}+{\left(\tau^{\prime \prime }\right)}^{2}}, & \tau^{\prime } & =\frac{P}{\sqrt{2}x_{1}x_{2}},\\ \tau^{\prime \prime } & =\frac{MR}{J}, & M & =P\left(L+\frac{x_{2}}{2}\right),\\ R & =\sqrt{\frac{x_{2}^{2}}{4}+{\left(\frac{x_{1}+x_{3}}{2}\right)}^{2}}, & J & =2\sqrt{2}x_{1}x_{2}\left[\frac{x_{2}^{2}}{4}+{\left(\frac{x_{1}+x_{3}}{2}\right)}^{2}\right],\\ \sigma (x) & =\frac{6PL}{x_{3}^{2}x_{4}}, & \delta (x) & =\frac{6PL^{3}}{Ex_{3}^{3}x_{4}},\\ P_{c}\left(x\right) & =\frac{4.0134E\sqrt{x_{3}^{2}x_{4}^{6}/36}}{L^{2}}\left(1-\frac{x_{3}}{2L}\sqrt{\frac{E}{4G}}\right). & & \end{array}$$

The functions τ(x), σ(x), δ(x), and $P_{c}\left(x\right)$ denote shear, bending, deflection, and buckling response. Constants are P=6000, L=14, $E=30\times 10^{6}$, $G=12\times 10^{6}$, $\tau_{\max}=13,600$, $\sigma_{\max}=30,000$, and $\delta_{\max}=0.25$. Bounds are $0.1\leq x_{1},x_{4}\leq 2$ and $0.1\leq x_{2},x_{3}\leq 10$.

Figure 8b shows average convergence, and Table 11 gives the 30-run comparison. WSAO reached Best and Avg cost 1.695217 with Std $7.36\times 10^{-8}$. Its mean tied for the leading rank, and the small variation indicates repeatable recovery of this design.

### 5.2. Tension/Compression Spring Design

**Figure 9:**
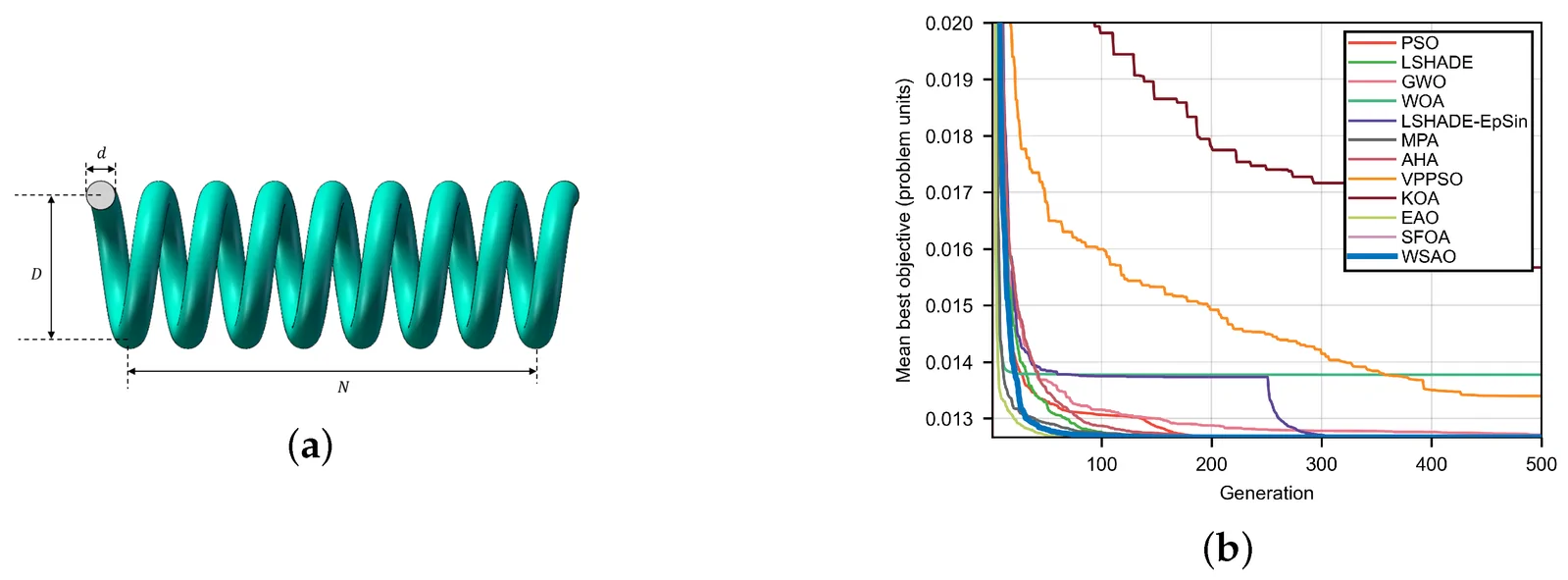
Tension/compression-spring case: (**a**) geometry and decision variables; (**b**) mean convergence histories across 30 runs. In panel (**b**), the horizontal axis is generation and the vertical axis is the mean best objective value in the problem-specific units defined above.

The spring case in Figure 9a minimizes weight under shear, surge-frequency, deflection, and geometric restrictions. Its continuous relaxation uses $x=\left[x_{1},x_{2},x_{3}\right]=\left[d,D,N_{c}\right]$. These entries are wire diameter, mean coil diameter, and relaxed active-coil count. Following [21],

(18) $$\min f\left(x\right)=\left(x_{3}+2\right)x_{2}x_{1}^{2},$$

where f(x) is spring weight. The constraints are

(19) $$\begin{array}{cc} g_{1}\left(x\right) & =1-\frac{x_{2}^{3}x_{3}}{71,785x_{1}^{4}}\leq 0,\\ g_{2}\left(x\right) & =\frac{4x_{2}^{2}-x_{1}x_{2}}{12,566(x_{2}x_{1}^{3}-x_{1}^{4})}+\frac{1}{5108x_{1}^{2}}-1\leq 0,\\ g_{3}\left(x\right) & =1-\frac{140.45x_{1}}{x_{2}^{2}x_{3}}\leq 0,\\ g_{4}\left(x\right) & =\frac{x_{1}+x_{2}}{1.5}-1\leq 0. \end{array}$$

Constraints $g_{1}$–$g_{4}$ represent shear stress, surge frequency, deflection, and geometry. Bounds are $0.05\leq x_{1}\leq 2$, $0.25\leq x_{2}\leq 1.3$, and $2\leq x_{3}\leq 15$.

Average convergence appears in Figure 9b, with final results in Table 12. WSAO found x=(0.051685,0.356683,11.288406) and weight 0.01266346. Avg was also 0.01266346, with Std $2.67\times 10^{-10}$. These values placed WSAO in the leading mean-rank group with negligible run-to-run variation.

### 5.3. Pressure Vessel Design

**Figure 10:**
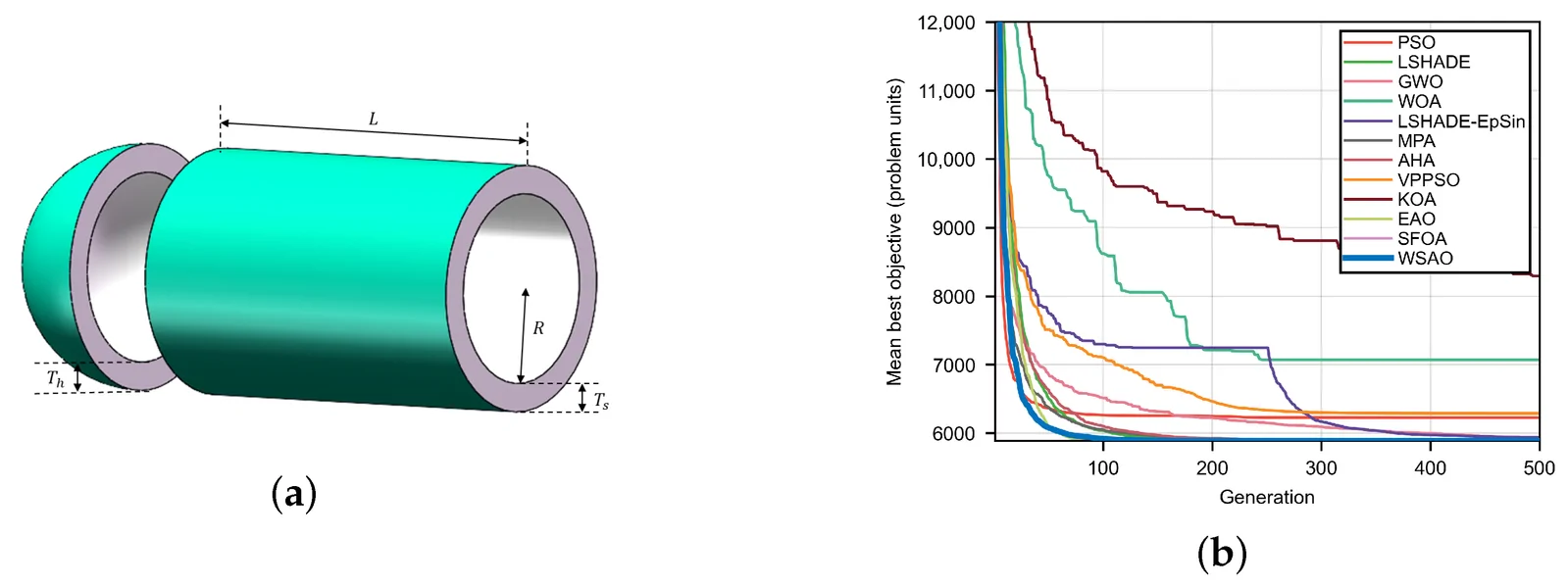
Pressure-vessel case: (**a**) geometry and decision variables; (**b**) mean convergence histories across 30 runs. In panel (**b**), the horizontal axis is generation and the vertical axis is the mean best objective value in the problem-specific units defined above.

The pressure vessel in Figure 10a minimizes manufacturing cost under thickness, volume, and length constraints. In $x=[x_{1},x_{2},x_{3},x_{4}]$, the first two entries are gauge-scaled thicknesses. The remaining entries are internal radius $R=x_{3}$ and cylindrical length $L=x_{4}$. Physical thicknesses are $T_{s}=0.0625x_{1}$ and $T_{h}=0.0625x_{2}$. Following [21],

(20) $$\min f\left(x\right)=0.6224T_{s}RL+1.7781T_{h}R^{2}+3.1661T_{s}^{2}L+19.84T_{s}^{2}R,$$

where f(x) is total manufacturing cost. The inequalities are

(21) $$\begin{array}{cc} g_{1}\left(x\right) & =-T_{s}+0.0193R\leq 0,\\ g_{2}\left(x\right) & =-T_{h}+0.00954R\leq 0,\\ g_{3}\left(x\right) & =-\pi R^{2}L-\frac{4}{3}\pi R^{3}+1,296,000\leq 0,\\ g_{4}\left(x\right) & =L-240\leq 0. \end{array}$$

Bounds are $0\leq x_{1},x_{2}\leq 99$ and 10≤R,L≤200. The stated gauge scaling connects these equations to the variables in Table 13.

Figure 10b and Table 13 report convergence and terminal performance. WSAO returned x=(12.450698,6.154387,40.319619,199.999997). Best and Avg costs were 5885.332788 and 5885.333222, respectively. The method ranked second by Avg, with Std $4.13\times 10^{-4}$ indicating stable performance near the leading value.

### 5.4. Three-Bar Truss Design

**Figure 11:**
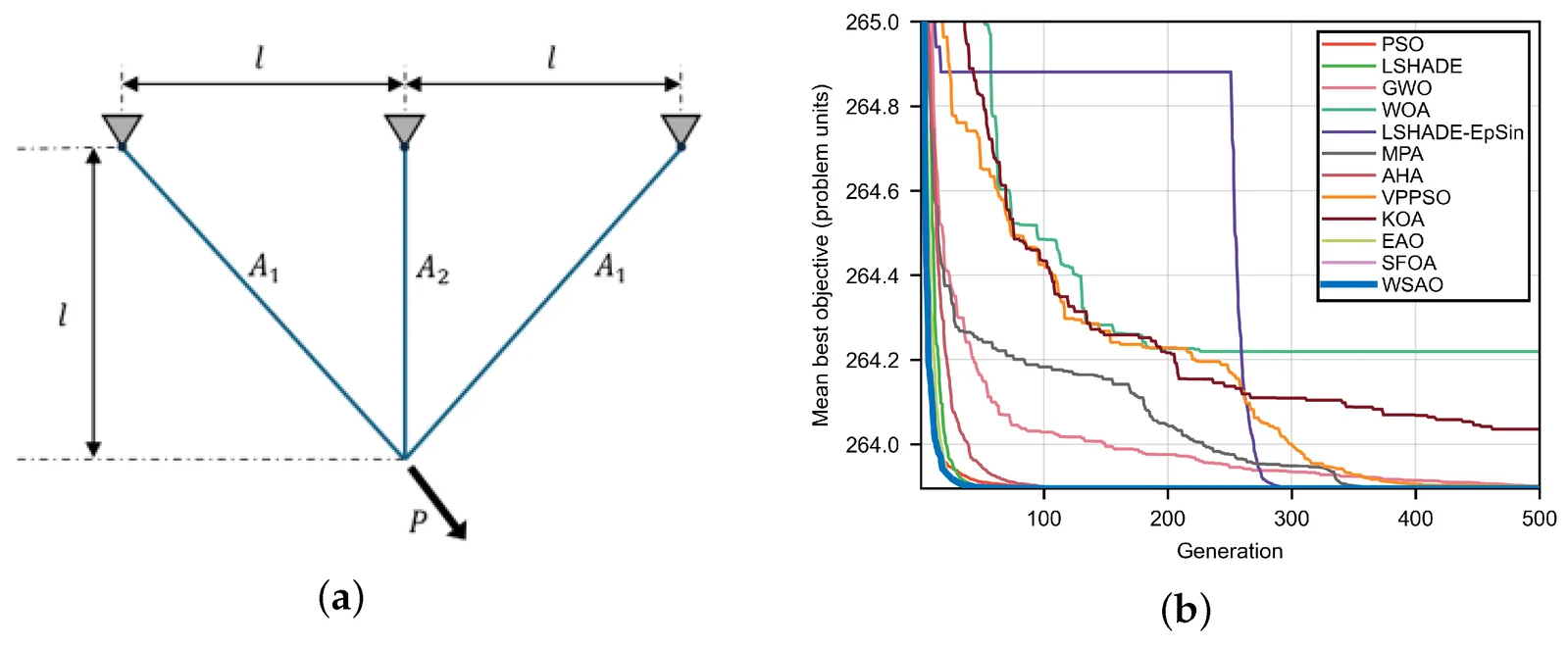
Three-bar-truss case: (**a**) geometry and decision variables; (**b**) mean convergence histories across 30 runs. In panel (**b**), the horizontal axis is generation and the vertical axis is the mean best objective value in the problem-specific units defined above.

The symmetric three-bar truss in Figure 11a minimizes volume under three stress limits. Symmetry imposes $A_{3}=A_{1}$, leaving $x=\left[x_{1},x_{2}\right]=\left[A_{1},A_{2}\right]$. Following [21],

(22) $$\min f\left(x\right)=\left(2\sqrt{2}x_{1}+x_{2}\right)l,$$

where f(x) is the truss volume and *l* is the member length. The stress constraints are

(23) $$\begin{array}{cc} g_{1}\left(x\right) & =\frac{\sqrt{2}x_{1}+x_{2}}{\sqrt{2}x_{1}^{2}+2x_{1}x_{2}}P-\sigma \leq 0,\\ g_{2}\left(x\right) & =\frac{x_{2}}{\sqrt{2}x_{1}^{2}+2x_{1}x_{2}}P-\sigma \leq 0,\\ g_{3}\left(x\right) & =\frac{P}{x_{1}+\sqrt{2}x_{2}}-\sigma \leq 0. \end{array}$$

The load is *P*, and the allowable stress is σ. The constants are l=100, P=2, and σ=2, with $0\leq x_{1},x_{2}\leq 1$.

Figure 11b shows mean convergence, and Table 14 lists the final designs. WSAO obtained x=(0.788675,0.408248) with Best and Avg volume 263.895843. Its Std was $2.05\times 10^{-11}$. WSAO therefore joined the leading rank group and reproduced the same design across runs.

### 5.5. Cantilever Beam Design

**Figure 12:**
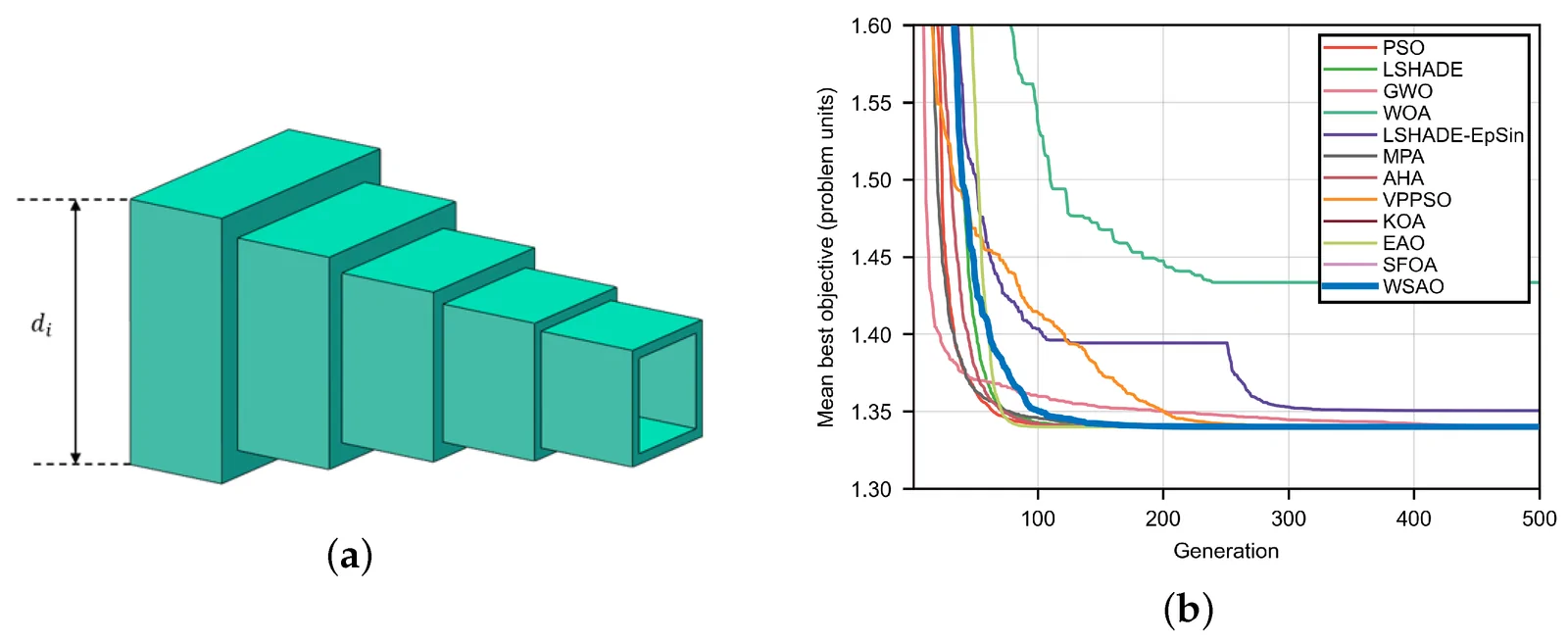
Cantilever-beam case: (**a**) geometry and decision variables; (**b**) mean convergence histories across 30 runs. In panel (**b**), the horizontal axis is generation and the vertical axis is the mean best objective value in the problem-specific units defined above.

The cantilever case in Figure 12a minimizes weight while satisfying one stiffness inequality. Five segment widths form $x=[x_{1},x_{2},x_{3},x_{4},x_{5}]$. Following the formulation in [21],

(24) $$\min f\left(x\right)=0.0624\left(x_{1}+x_{2}+x_{3}+x_{4}+x_{5}\right),$$

where f(x) is structural weight. The constraint is

(25) $$g\left(x\right)=\frac{61}{x_{1}^{3}}+\frac{37}{x_{2}^{3}}+\frac{19}{x_{3}^{3}}+\frac{7}{x_{4}^{3}}+\frac{1}{x_{5}^{3}}-1\leq 0,$$

where g(x) measures the stiffness violation. Each variable satisfies $0.01\leq x_{i}\leq 100$ for i=1,…,5.

Figure 12b presents mean convergence, and Table 15 compares final outcomes. WSAO found x=(6.015571,5.309530,4.494801,3.501669,2.152090). The best and Avg weight were 1.339956, with Std $2.56\times 10^{-8}$. These values placed WSAO in the leading group with stable final quality.

Across the five cases, WSAO shared or held the leading Avg rank for welded beam, spring, truss, and cantilever design. It ranked second for the pressure vessel. Low Std values across all cases indicate consistent terminal quality under several dimensions, objectives, and constraint structures.

## 6. Discussion

### 6.1. Interpretation of the Observed Performance

WSAO achieved the lowest Friedman mean rank in both CEC suites and joined the leading Avg group in four constrained cases. Convergence traces, trajectories, and targeted controls identify three mechanisms associated with this performance:

1. **Probabilistic strategy switching.** MPA and the Sinh Cosh Optimizer (SCHO) change structures through iteration-dependent rules, whereas WSAO can revisit any state through correlated Markov transitions. Empirical occupancy and episode lengths agreed with P. No alternative matrix improved all selected functions, although persistence affected F1. The fixed matrix is therefore supported as a reproducible baseline, not a universal optimum.
2. **Multi-center exploitation.** Several nests support simultaneous refinement around separated elite regions. One nest worsened F4, F7, and F12 but improved F1. Concurrent centers therefore helped the selected structured landscapes but were unnecessary for the unimodal case. Algorithm 1 enforces separation during primary screening and relaxes it only when the fallback branch fills missing nests.
3. **Complementary perturbation.** Global and differential trials add domain-wide and population-difference proposals after each weather update. Their budget-matched removal worsened all selected functions, the most consistent ablation effect. Eliminating rainy displacement damping also worsened F1 without a corrected improvement elsewhere.

### 6.2. Scope and Future Extensions

The evidence supports continuous single-objective use at D=10 and motivates six bounded extensions:

1. **Adaptive transition models.** The fixed P is a controlled reference. Future variants could update transition probabilities from diversity or improvement signals while retaining the same states.
2. **Landscape-aware component control.** Temporal dependence and nest count had function-specific effects. Diversity measures could regulate persistence or active centers, using the current configuration as the reference.
3. **Scale and computational profiling.** The present 41-function comparison fixes D=10 and a nominal 50,000-evaluation budget. Higher dimensions and timing studies are needed to measure archive and nest scaling.
4. **Broader constraint handling.** Static penalties produced competitive results in five cases. Feasibility rules, adaptive penalties, or repair operators could address tighter or application-specific constraints.
5. **Multi-objective and discrete extensions.** The separation between controller and operators permits future Pareto selection, binary encodings, or combinatorial neighborhoods. These extensions require independent validation.
6. **Theoretical characterization.** The explicit matrix permits analysis of occupancy and recurrence. Specialized derivative-free root solvers obtain local convergence orders under smoothness and initialization assumptions [55]. For WSAO, convergence in probability should instead be studied under bounded-domain, archive, and state-visitation assumptions.

### 6.3. Implications for Metaheuristic Algorithm Design

WSAO replaces a fixed phase order with persistent but recurrent state selection. The transition model and state-specific equations remain separate modules. Consequently, either module can be modified without redefining the other. Feedback-driven transition probabilities are one possible extension, but their benefit remains to be tested.

## 7. Conclusions

The Weather State Ants Optimizer uses a discrete-time Markov chain to recurrently select global exploration, nest congregation, or local refinement. A dynamic archive supplies several elite regions as concurrent candidate centers.

At D=10, WSAO achieved the lowest Friedman mean ranks among 12 algorithms: 2.48 on CEC2017 and 2.33 on CEC2022. It placed within the top three on 25 of 29 and 10 of 12 functions, respectively. In constrained design, WSAO joined the leading Avg group on four cases and ranked second for the pressure vessel. Variation across runs remained low.

In targeted controls, removing the trial displacement increased error on all five selected CEC2022 functions. Multiple nests helped the tested structured cases but not unimodal F1. No alternative transition matrix improved every selected function. Rainy damping also avoided the F1 deterioration observed at $\eta_{r}=1.0$. These results support the integrated design while limiting component claims to the tested conditions. Further work should evaluate feedback-controlled transitions, higher dimensions, bounded archives, and non-continuous problem classes.

## Figures and Tables

**Table 1:** Structural comparison between WSAO and representative metaheuristic designs. Bold identifies the proposed method and its defining switching mechanism.

Algorithm	Variable Structure	Probabilistic Switching	Multi-Center Exploitation	Switching Mechanism
PSO [16]	×	×	×	—
GWO [45]	×	×	×	—
WOA [48]	×	√ ^P^	×	Parameter-based
MPA [8]	√	×	×	Iteration-based
SCHO [9]	√	×	×	Trigonometric
**WSAO (Ours)**	√	√ ^M^	√	**Markov process**

^P^ Parameter adaptation; ^M^ Markov-driven probabilistic switching

**Table 2:** WSAO settings applied throughout the reported experiments.

Component	Parameter	Setting
Weather-state controller	Transition matrix P	Equation (3)
Nest system	Coverage coefficient *k*	0.4
Nest system	Target number of nests $n_{\mathrm{Nest}}$	5
Sunny state	Inertia weight $w^{\left(t\right)}$	Linear decrease from 0.9 to 0.1
Sunny state	Cognitive coefficient $c_{1}$	1.6
Sunny state	Social coefficient $c_{2}^{\left(t\right)}$	Linear increase from 0.4 to 1.6
Cloudy state	$w^{\left(t\right)}$, $c_{1c}$, and $c_{3c}$	0.9−0.8t/T, 0.6, and 2.0
Rainy state	$w^{\left(t\right)}$, $c_{1r}$, $c_{3r}$, and $\eta_{r}$	0.9−0.8t/T, 1.6, 1.6, and 0.5
Trial perturbation	$c_{\ell 1}^{\left(t\right)}$ and $c_{\ell 2}$	${\left(1-t/T\right)}^{2t/T}$ and 0.2
Trial perturbation	Global-branch probability	0.2

**Table 3:** Control settings adopted for the 11 comparator algorithms.

Algorithm	Year	Parameter	Value	Algorithm	Year	Parameter	Value
PSO	1995	Inertia weight *w*	0.5	AHA	2022	*M*	2n
		$c_{1}$	1.5				
		$c_{2}$	1.5				
LSHADE	2014	Memory size	5	VPPSO	2023	α	0.3
		Pbest size	5			$N_{1}$	40
		*H*	6			$N_{2}$	60
LSHADE-EpSin	2016	$Freq_{0}$	0.5	KOA	2023	*T*	3
		$P_{b}$	0.4			$\mu_{0}$	0.1
		$P_{s}$	0.5			γ	15
WOA	2016	*a*	[2,0]	SFOA	2025	*C*	0.8
		Spiral factor *b*	1				
MPA	2020	FADs	0.2	EAO	2025	EC	0.1
		*P*	0.5				
GWO	2014	*a*	[2,0]				

**Table 4:** Targeted Markov-matrix sensitivity and component-ablation results on representative CEC2022 functions. Entries are median paired changes in $\log_{10}\left(\mathrm{error}+10^{-12}\right)$ relative to Full WSAO; negative values favor the listed variant. Bold entries remain significant after Holm correction within the corresponding experiment family. W / L / T denotes wins / losses / ties for the listed variant against Full WSAO across all 150 paired runs.

Variant	F1	F4	F7	F10	F12	W / L / T
Low-persistence matrix	**+0.694**	+0.014	+0.102	−$1.48\times 10^{-5}$	−$4.06\times 10^{-7}$	63 / 84 / 3
High-persistence matrix	**−0.738**	+0.015	−0.037	−$2.18\times 10^{-5}$	+$4.04\times 10^{-5}$	79 / 69 / 2
Strong-recovery matrix	+0.296	−0.112	−0.318	−$3.22\times 10^{-5}$	−$5.78\times 10^{-13}$	84 / 64 / 2
Independent state sampling	**+0.706**	−0.047	−0.092	+$1.04\times 10^{-5}$	+$1.56\times 10^{-5}$	63 / 87 / 0
Single nest	**−6.105**	**+0.140**	**+0.687**	+$3.73\times 10^{-5}$	**+0.006**	62 / 86 / 2
No trial perturbation	**+3.682**	**+0.172**	**+0.691**	+$2.83\times 10^{-4}$	**+0.010**	14 / 136 / 0
$\eta_{r}=1.0$	**+1.037**	−0.015	−0.056	+$1.31\times 10^{-4}$	−0.002	57 / 90 / 3

**Table 5:** Suite-level Friedman ordering on CEC2017: D=10, 29 functions, and 30 runs per function.

Algorithm	Mean Rank	Final Rank
**WSAO**	**2.48**	**1**
LSHADE-EpSin	2.50	2
MPA	2.79	3
EAO	3.12	4
LSHADE	5.79	5
VPPSO	6.90	6
GWO	6.90	7
AHA	8.03	8
PSO	8.17	9
WOA	9.41	10
KOA	10.93	11
SFOA	10.97	12

**Table 6:** Complete CEC2017 results at D=10 over 30 independent runs. For each function, the best Avg value and its Std and Rank are shown in bold; the proposed method name is also bold.

**Algorithm**	**F1**			**F3**			**F4**			**F5**			**F6**			**F7**		
	**Avg**	**Std**	**Rank**	**Avg**	**Std**	**Rank**	**Avg**	**Std**	**Rank**	**Avg**	**Std**	**Rank**	**Avg**	**Std**	**Rank**	**Avg**	**Std**	**Rank**
**WSAO**	100.014	$2.55\times 10^{-2}$	3	**300.000**	$2.94\times 10^{-8}$	**1**	**400.000**	$2.21\times 10^{-4}$	**1**	505.838	$2.57\times 10^{0}$	2	600.006	$3.90\times 10^{-3}$	5	716.140	$4.02\times 10^{0}$	2
PSO	$2.92\times 10^{8}$	$2.60\times 10^{8}$	10	824.120	$5.77\times 10^{2}$	6	457.870	$4.25\times 10^{1}$	9	538.839	$1.50\times 10^{1}$	9	620.399	$7.83\times 10^{0}$	9	745.487	$1.56\times 10^{1}$	8
LSHADE	$2.03\times 10^{4}$	$8.51\times 10^{4}$	6	4651.017	$5.05\times 10^{3}$	10	405.705	$1.48\times 10^{0}$	6	514.755	$9.44\times 10^{0}$	5	600.004	$1.56\times 10^{-2}$	4	731.660	$8.83\times 10^{0}$	6
GWO	$2.26\times 10^{7}$	$8.04\times 10^{7}$	8	1127.321	$1.39\times 10^{3}$	7	414.213	$1.83\times 10^{1}$	7	516.033	$1.08\times 10^{1}$	6	600.505	$6.44\times 10^{-1}$	6	730.652	$1.06\times 10^{1}$	5
WOA	$1.88\times 10^{6}$	$3.92\times 10^{6}$	7	1222.002	$6.54\times 10^{2}$	8	440.255	$5.10\times 10^{1}$	8	545.940	$2.05\times 10^{1}$	10	635.894	$1.36\times 10^{1}$	11	775.971	$3.09\times 10^{1}$	10
LS-EpSin	**100.000**	$0.00\times 10^{0}$	**1**	**300.000**	$0.00\times 10^{0}$	**1**	**400.000**	$1.98\times 10^{-13}$	**1**	**503.192**	$9.73\times 10^{-1}$	**1**	**600.000**	$4.35\times 10^{-7}$	**1**	**713.452**	$9.58\times 10^{-1}$	**1**
MPA	116.320	$1.90\times 10^{1}$	4	300.000	$7.67\times 10^{-6}$	4	400.113	$2.71\times 10^{-1}$	4	508.981	$2.56\times 10^{0}$	4	600.003	$6.74\times 10^{-3}$	3	719.621	$3.11\times 10^{0}$	3
AHA	$2.17\times 10^{8}$	$1.03\times 10^{9}$	9	4595.192	$6.36\times 10^{3}$	9	715.899	$1.06\times 10^{3}$	12	527.870	$2.47\times 10^{1}$	8	607.446	$1.47\times 10^{1}$	8	762.981	$3.33\times 10^{1}$	9
VPPSO	2120.514	$2.26\times 10^{3}$	5	305.701	$1.63\times 10^{1}$	5	404.679	$2.17\times 10^{0}$	5	523.600	$7.94\times 10^{0}$	7	605.858	$5.17\times 10^{0}$	7	737.344	$1.36\times 10^{1}$	7
KOA	$4.07\times 10^{9}$	$1.14\times 10^{9}$	12	$1.35\times 10^{4}$	$2.78\times 10^{3}$	11	669.224	$7.76\times 10^{1}$	11	578.146	$8.93\times 10^{0}$	12	644.764	$5.05\times 10^{0}$	12	890.789	$2.78\times 10^{1}$	12
EAO	**100.000**	$3.73\times 10^{-15}$	**1**	**300.000**	$0.00\times 10^{0}$	**1**	**400.000**	$1.06\times 10^{-14}$	**1**	508.797	$5.37\times 10^{0}$	3	600.000	$9.70\times 10^{-6}$	2	729.299	$7.55\times 10^{0}$	4
SFOA	$2.57\times 10^{9}$	$2.90\times 10^{9}$	11	$2.61\times 10^{4}$	$1.63\times 10^{4}$	12	609.532	$1.47\times 10^{2}$	10	573.466	$2.17\times 10^{1}$	11	635.317	$1.14\times 10^{1}$	10	841.436	$5.74\times 10^{1}$	11
**Algorithm**	**F8**			**F9**			**F10**			**F11**			**F12**			**F13**		
	**Avg**	**Std**	**Rank**	**Avg**	**Std**	**Rank**	**Avg**	**Std**	**Rank**	**Avg**	**Std**	**Rank**	**Avg**	**Std**	**Rank**	**Avg**	**Std**	**Rank**
**WSAO**	806.587	$2.52\times 10^{0}$	2	**900.000**	$3.61\times 10^{-8}$	**1**	1205.287	$1.53\times 10^{2}$	2	1101.523	$1.12\times 10^{0}$	3	1327.441	$7.36\times 10^{1}$	3	1309.024	$3.70\times 10^{0}$	4
PSO	820.769	$8.24\times 10^{0}$	7	1031.68	$9.18\times 10^{1}$	8	1932.859	$2.49\times 10^{2}$	8	1174.737	$4.35\times 10^{1}$	8	$6.33\times 10^{5}$	$1.71\times 10^{6}$	6	3367.102	$3.15\times 10^{3}$	7
LSHADE	818.343	$8.61\times 10^{0}$	6	900.023	$1.28\times 10^{-1}$	4	2091.417	$3.57\times 10^{2}$	10	1106.323	$2.73\times 10^{0}$	5	$4.51\times 10^{5}$	$6.74\times 10^{5}$	5	1333.942	$4.52\times 10^{1}$	5
GWO	811.706	$4.67\times 10^{0}$	5	906.476	$1.18\times 10^{1}$	6	1482.948	$2.88\times 10^{2}$	4	1133.902	$3.15\times 10^{1}$	6	$7.52\times 10^{5}$	$1.68\times 10^{6}$	7	$1.33\times 10^{4}$	$7.60\times 10^{3}$	8
WOA	841.758	$1.94\times 10^{1}$	10	1291.23	$2.75\times 10^{2}$	9	2073.254	$2.93\times 10^{2}$	9	1198.116	$7.33\times 10^{1}$	9	$4.06\times 10^{6}$	$4.62\times 10^{6}$	9	$1.92\times 10^{4}$	$1.54\times 10^{4}$	10
LS-EpSin	**803.075**	** $8.88\times 10^{-1}$ **	**1**	**900.000**	$0.00\times 10^{0}$	**1**	**1149.516**	$9.56\times 10^{1}$	**1**	**1100.255**	$6.79\times 10^{-1}$	**1**	1382.404	$1.14\times 10^{2}$	4	1303.540	$2.86\times 10^{0}$	2
MPA	808.557	$3.01\times 10^{0}$	3	900.073	$1.96\times 10^{-1}$	5	1353.690	$1.28\times 10^{2}$	3	1102.681	$1.46\times 10^{0}$	4	**1273.471**	$5.88\times 10^{1}$	**1**	1307.413	$2.39\times 10^{0}$	3
AHA	831.623	$2.39\times 10^{1}$	9	1328.41	$5.46\times 10^{2}$	10	1577.807	$2.53\times 10^{2}$	5	$4.34\times 10^{6}$	$1.65\times 10^{7}$	12	$7.18\times 10^{7}$	$3.93\times 10^{8}$	11	1416.024	$2.19\times 10^{2}$	6
VPPSO	821.292	$6.38\times 10^{0}$	8	917.151	$2.14\times 10^{1}$	7	1762.505	$3.53\times 10^{2}$	6	1140.880	$2.21\times 10^{1}$	7	$1.20\times 10^{6}$	$1.21\times 10^{6}$	8	$1.36\times 10^{4}$	$1.10\times 10^{4}$	9
KOA	872.506	$1.07\times 10^{1}$	11	1918.21	$2.21\times 10^{2}$	11	2522.290	$1.74\times 10^{2}$	11	1516.800	$1.44\times 10^{2}$	10	$1.11\times 10^{8}$	$7.50\times 10^{7}$	12	$6.26\times 10^{5}$	$6.59\times 10^{5}$	11
EAO	809.971	$6.49\times 10^{0}$	4	**900.000**	$0.00\times 10^{0}$	**1**	1884.883	$4.09\times 10^{2}$	7	1100.455	$7.74\times 10^{-1}$	2	1319.735	$7.85\times 10^{1}$	2	**1303.471**	$2.97\times 10^{0}$	**1**
SFOA	874.354	$2.32\times 10^{1}$	12	1930.43	$8.46\times 10^{2}$	12	2724.235	$2.39\times 10^{2}$	12	1561.058	$3.33\times 10^{2}$	11	$6.62\times 10^{7}$	$9.00\times 10^{7}$	10	$1.42\times 10^{6}$	$3.97\times 10^{6}$	12
**Algorithm**	**F14**			**F15**			**F16**			**F17**			**F18**			**F19**		
	**Avg**	**Std**	**Rank**	**Avg**	**Std**	**Rank**	**Avg**	**Std**	**Rank**	**Avg**	**Std**	**Rank**	**Avg**	**Std**	**Rank**	**Avg**	**Std**	**Rank**
**WSAO**	**1402.540**	$1.59\times 10^{0}$	**1**	1501.785	$6.59\times 10^{-1}$	4	**1601.380**	$9.44\times 10^{-1}$	**1**	1711.525	$9.25\times 10^{0}$	3	**1806.594**	$5.63\times 10^{0}$	**1**	1901.640	$6.35\times 10^{-1}$	4
PSO	1471.826	$2.53\times 10^{1}$	7	1689.051	$9.91\times 10^{1}$	6	1806.541	$1.33\times 10^{2}$	8	1774.917	$3.96\times 10^{1}$	9	4675.158	$3.60\times 10^{3}$	6	1968.572	$1.34\times 10^{2}$	7
LSHADE	1422.286	$4.37\times 10^{0}$	6	1504.222	$2.38\times 10^{0}$	5	1611.459	$1.26\times 10^{1}$	5	1726.977	$6.37\times 10^{0}$	4	1825.060	$8.57\times 10^{0}$	5	1902.373	$8.78\times 10^{-1}$	6
GWO	3095.269	$1.82\times 10^{3}$	11	2682.804	$1.41\times 10^{3}$	7	1684.226	$9.31\times 10^{1}$	6	1748.289	$1.60\times 10^{1}$	7	$2.41\times 10^{4}$	$1.44\times 10^{4}$	8	8785.964	$5.75\times 10^{3}$	9
WOA	1913.462	$1.11\times 10^{3}$	9	5220.424	$2.97\times 10^{3}$	9	1849.516	$1.30\times 10^{2}$	9	1790.955	$4.33\times 10^{1}$	10	$1.72\times 10^{4}$	$1.23\times 10^{4}$	7	$3.22\times 10^{4}$	$4.30\times 10^{4}$	11
LS-EpSin	1404.718	$8.11\times 10^{0}$	2	**1500.294**	$2.48\times 10^{-1}$	**1**	1601.394	$4.53\times 10^{-1}$	2	**1701.756**	$1.16\times 10^{0}$	**1**	1808.834	$9.78\times 10^{0}$	2	**1900.313**	** $6.32\times 10^{-1}$ **	**1**
MPA	1405.309	$3.91\times 10^{0}$	3	1501.306	$8.01\times 10^{-1}$	3	1602.330	$2.23\times 10^{0}$	3	1710.285	$1.01\times 10^{1}$	2	1809.093	$4.45\times 10^{0}$	3	1901.710	$6.59\times 10^{-1}$	5
AHA	1408.168	$8.89\times 10^{0}$	5	$2.57\times 10^{7}$	$1.40\times 10^{8}$	12	1885.286	$2.87\times 10^{2}$	10	1728.511	$1.72\times 10^{1}$	5	$4.53\times 10^{8}$	$1.72\times 10^{9}$	12	1901.563	$2.16\times 10^{0}$	3
VPPSO	1614.687	$3.20\times 10^{2}$	8	3811.987	$1.76\times 10^{3}$	8	1750.256	$1.11\times 10^{2}$	7	1773.142	$2.88\times 10^{1}$	8	$2.42\times 10^{4}$	$1.42\times 10^{4}$	9	5969.985	$5.34\times 10^{3}$	8
KOA	2024.430	$4.56\times 10^{2}$	10	5900.096	$2.74\times 10^{3}$	10	1964.410	$9.13\times 10^{1}$	11	1865.429	$3.36\times 10^{1}$	11	$1.39\times 10^{6}$	$1.08\times 10^{6}$	10	$1.11\times 10^{4}$	$1.15\times 10^{4}$	10
EAO	1407.125	$8.30\times 10^{0}$	4	1500.456	$3.48\times 10^{-1}$	2	1604.245	$4.57\times 10^{0}$	4	1737.702	$4.74\times 10^{0}$	6	1812.409	$9.48\times 10^{0}$	4	1900.829	$5.20\times 10^{-1}$	2
SFOA	9326.138	$9.18\times 10^{3}$	12	$1.95\times 10^{4}$	$1.31\times 10^{4}$	11	1982.030	$1.54\times 10^{2}$	12	1872.891	$8.78\times 10^{1}$	12	$1.69\times 10^{6}$	$3.00\times 10^{6}$	11	$3.56\times 10^{4}$	$3.82\times 10^{4}$	12
**Algorithm**	**F20**			**F21**			**F22**			**F23**			**F24**			**F25**		
	**Avg**	**Std**	**Rank**	**Avg**	**Std**	**Rank**	**Avg**	**Std**	**Rank**	**Avg**	**Std**	**Rank**	**Avg**	**Std**	**Rank**	**Avg**	**Std**	**Rank**
**WSAO**	2003.208	$5.08\times 10^{0}$	2	**2200.000**	$1.45\times 10^{-5}$	**1**	2277.561	$4.35\times 10^{1}$	2	2607.862	$2.56\times 10^{0}$	3	2656.813	$1.13\times 10^{2}$	3	2900.871	$1.16\times 10^{1}$	2
PSO	2119.696	$6.16\times 10^{1}$	9	2277.745	$6.23\times 10^{1}$	9	2337.375	$2.18\times 10^{1}$	8	2681.265	$3.45\times 10^{1}$	11	2741.051	$1.23\times 10^{2}$	7	2950.408	$2.57\times 10^{1}$	9
LSHADE	2014.665	$8.62\times 10^{0}$	3	2287.384	$5.34\times 10^{1}$	10	2301.274	$1.72\times 10^{0}$	6	2626.581	$9.46\times 10^{0}$	8	2749.360	$3.22\times 10^{1}$	9	2917.979	$2.22\times 10^{1}$	5
GWO	2052.926	$3.19\times 10^{1}$	6	2293.375	$4.75\times 10^{1}$	11	2305.985	$4.70\times 10^{0}$	7	2619.201	$9.93\times 10^{0}$	6	2728.827	$6.16\times 10^{1}$	6	2940.119	$1.35\times 10^{1}$	7
WOA	2167.306	$7.91\times 10^{1}$	11	2331.938	$4.27\times 10^{1}$	12	2350.939	$2.06\times 10^{2}$	9	2649.144	$2.05\times 10^{1}$	9	2765.889	$6.57\times 10^{1}$	11	2943.197	$2.92\times 10^{1}$	8
LS-EpSin	**2000.276**	$1.54\times 10^{-1}$	**1**	2271.634	$3.85\times 10^{1}$	8	2300.112	$1.77\times 10^{-1}$	5	2604.569	$1.27\times 10^{0}$	2	2699.222	$3.64\times 10^{1}$	4	2925.353	$2.28\times 10^{1}$	6
MPA	2017.545	$9.95\times 10^{0}$	5	2200.007	$3.70\times 10^{-2}$	2	**2251.085**	$4.57\times 10^{1}$	**1**	**2600.137**	$5.68\times 10^{1}$	**1**	**2500.002**	$2.26\times 10^{-3}$	**1**	**2867.981**	$9.08\times 10^{1}$	**1**
AHA	2100.502	$2.14\times 10^{2}$	8	2239.665	$6.27\times 10^{1}$	4	2497.744	$5.99\times 10^{2}$	10	2609.224	$6.01\times 10^{1}$	5	2632.520	$2.14\times 10^{2}$	2	3064.128	$2.57\times 10^{2}$	10
VPPSO	2093.741	$4.96\times 10^{1}$	7	2266.919	$5.90\times 10^{1}$	7	2298.190	$1.74\times 10^{1}$	4	2620.154	$8.10\times 10^{0}$	7	2742.680	$4.67\times 10^{1}$	8	2913.209	$2.22\times 10^{1}$	4
KOA	2165.230	$3.30\times 10^{1}$	10	2261.184	$2.21\times 10^{1}$	6	2567.218	$1.08\times 10^{2}$	12	2694.370	$2.13\times 10^{1}$	12	2757.221	$4.68\times 10^{1}$	10	3134.951	$6.77\times 10^{1}$	12
EAO	2015.373	$1.11\times 10^{1}$	4	2203.609	$1.98\times 10^{1}$	3	2282.018	$3.81\times 10^{1}$	3	2608.274	$3.25\times 10^{0}$	4	2719.286	$5.97\times 10^{1}$	5	2901.046	$1.15\times 10^{1}$	3
SFOA	2202.878	$8.17\times 10^{1}$	12	2256.750	$2.50\times 10^{1}$	5	2547.000	$1.90\times 10^{2}$	11	2670.898	$1.54\times 10^{1}$	10	2782.907	$5.87\times 10^{1}$	12	3089.642	$1.44\times 10^{2}$	11
**Algorithm**	**F26**			**F27**			**F28**			**F29**			**F30**					
	**Avg**	**Std**	**Rank**	**Avg**	**Std**	**Rank**	**Avg**	**Std**	**Rank**	**Avg**	**Std**	**Rank**	**Avg**	**Std**	**Rank**			
**WSAO**	2876.667	$7.74\times 10^{1}$	2	3072.742	$5.67\times 10^{-1}$	2	3203.920	$8.38\times 10^{1}$	3	3141.763	$4.25\times 10^{0}$	2	**3205.926**	$7.90\times 10^{-1}$	**1**			
PSO	3233.898	$2.14\times 10^{2}$	9	3174.783	$3.28\times 10^{1}$	12	3346.110	$1.47\times 10^{2}$	9	3263.441	$6.44\times 10^{1}$	9	$3.30\times 10^{5}$	$5.92\times 10^{5}$	7			
LSHADE	2952.139	$9.50\times 10^{1}$	7	**3071.843**	$1.06\times 10^{0}$	**1**	3272.552	$2.78\times 10^{-1}$	5	3198.264	$2.72\times 10^{1}$	7	3512.056	$4.83\times 10^{2}$	4			
GWO	2949.033	$1.66\times 10^{2}$	6	3094.506	$3.52\times 10^{0}$	6	3322.045	$1.06\times 10^{2}$	7	3183.151	$3.89\times 10^{1}$	6	$6.09\times 10^{5}$	$7.29\times 10^{5}$	9			
WOA	3333.621	$4.88\times 10^{2}$	10	3134.966	$4.35\times 10^{1}$	10	3370.465	$2.12\times 10^{2}$	10	3305.404	$8.08\times 10^{1}$	10	$5.53\times 10^{5}$	$8.88\times 10^{5}$	8			
LS-EpSin	2900.000	$1.89\times 10^{-13}$	3	3091.407	$2.80\times 10^{0}$	5	3245.630	$1.52\times 10^{2}$	4	**3134.646**	$4.06\times 10^{0}$	**1**	$7.23\times 10^{4}$	$2.68\times 10^{5}$	5			
MPA	**2733.866**	$1.16\times 10^{2}$	**1**	3089.305	$3.20\times 10^{-1}$	3	**3080.001**	$7.61\times 10^{1}$	**1**	3142.341	$6.38\times 10^{0}$	3	3448.164	$5.10\times 10^{1}$	3			
AHA	3057.567	$5.21\times 10^{2}$	8	3099.541	$1.10\times 10^{1}$	8	3289.129	$2.38\times 10^{2}$	6	3174.390	$3.26\times 10^{1}$	5	$2.80\times 10^{7}$	$9.39\times 10^{7}$	12			
VPPSO	2920.907	$2.26\times 10^{2}$	5	3098.206	$1.84\times 10^{1}$	7	3340.439	$1.61\times 10^{2}$	8	3212.841	$4.20\times 10^{1}$	8	$2.41\times 10^{5}$	$4.30\times 10^{5}$	6			
KOA	3406.708	$1.12\times 10^{2}$	12	3163.974	$1.31\times 10^{1}$	11	3418.437	$7.47\times 10^{1}$	12	3348.096	$4.86\times 10^{1}$	11	$5.42\times 10^{6}$	$2.66\times 10^{6}$	11			
EAO	2900.000	$1.46\times 10^{-13}$	3	3089.504	$2.11\times 10^{-1}$	4	3100.000	$2.39\times 10^{-13}$	2	3145.590	$9.72\times 10^{0}$	4	3428.943	$4.42\times 10^{1}$	2			
SFOA	3371.589	$3.04\times 10^{2}$	11	3109.795	$8.53\times 10^{0}$	9	3411.556	$1.33\times 10^{2}$	11	3359.993	$1.10\times 10^{2}$	12	$7.78\times 10^{5}$	$2.36\times 10^{5}$	10			

**Table 7:** Holm-adjusted two-sided Wilcoxon rank-sum test *p*-values comparing WSAO with each competitor on CEC2017 at D=10. CEC2017 and CEC2022 were treated as separate test families; bold values indicate $p_{\mathrm{adj}}<0.05$.

Algorithm	F1	F3	F4	F5	F6	F7
PSO	$9.084\times 10^{-9}$	$9.084\times 10^{-9}$	$9.084\times 10^{-9}$	$9.084\times 10^{-9}$	$9.084\times 10^{-9}$	$9.084\times 10^{-9}$
LSHADE	$2.893\times 10^{-8}$	$9.084\times 10^{-9}$	$9.084\times 10^{-9}$	$5.307\times 10^{-4}$	$1.647\times 10^{-6}$	$2.316\times 10^{-7}$
GWO	$9.084\times 10^{-9}$	$9.084\times 10^{-9}$	$9.084\times 10^{-9}$	$5.656\times 10^{-5}$	$9.084\times 10^{-9}$	$5.462\times 10^{-7}$
WOA	$9.084\times 10^{-9}$	$9.084\times 10^{-9}$	$9.084\times 10^{-9}$	$9.084\times 10^{-9}$	$9.084\times 10^{-9}$	$9.084\times 10^{-9}$
LS-EpSin	$3.866\times 10^{-10}$	$3.866\times 10^{-10}$	$2.001\times 10^{-9}$	$7.871\times 10^{-4}$	$9.084\times 10^{-9}$	$2.391\times 10^{-2}$
MPA	$9.084\times 10^{-9}$	$9.084\times 10^{-9}$	$9.084\times 10^{-9}$	$9.084\times 10^{-9}$	$9.084\times 10^{-9}$	$9.084\times 10^{-9}$
AHA	$9.084\times 10^{-9}$	$9.084\times 10^{-9}$	$9.084\times 10^{-9}$	$3.696\times 10^{-8}$	$1.000\times 10^{0}$	$9.084\times 10^{-9}$
VPPSO	$9.084\times 10^{-9}$	$1.000\times 10^{0}$	$9.084\times 10^{-9}$	$3.158\times 10^{-8}$	$9.084\times 10^{-9}$	$3.158\times 10^{-8}$
KOA	$9.084\times 10^{-9}$	$9.084\times 10^{-9}$	$9.084\times 10^{-9}$	$9.084\times 10^{-9}$	$9.084\times 10^{-9}$	$9.084\times 10^{-9}$
EAO	$7.399\times 10^{-10}$	$3.866\times 10^{-10}$	$5.402\times 10^{-10}$	$1.000\times 10^{0}$	$8.737\times 10^{-9}$	$2.804\times 10^{-6}$
SFOA	$9.084\times 10^{-9}$	$9.084\times 10^{-9}$	$9.084\times 10^{-9}$	$9.084\times 10^{-9}$	$9.084\times 10^{-9}$	$9.084\times 10^{-9}$
Algorithm	F8	F9	F10	F11	F12	F13
PSO	$9.901\times 10^{-9}$	$9.084\times 10^{-9}$	$9.084\times 10^{-9}$	$9.084\times 10^{-9}$	$9.084\times 10^{-9}$	$9.084\times 10^{-9}$
LSHADE	$1.054\times 10^{-7}$	$1.000\times 10^{0}$	$9.084\times 10^{-9}$	$2.065\times 10^{-8}$	$3.696\times 10^{-8}$	$5.679\times 10^{-6}$
GWO	$9.481\times 10^{-4}$	$9.084\times 10^{-9}$	$2.048\times 10^{-3}$	$9.084\times 10^{-9}$	$9.084\times 10^{-9}$	$9.084\times 10^{-9}$
WOA	$9.084\times 10^{-9}$	$9.084\times 10^{-9}$	$9.084\times 10^{-9}$	$9.084\times 10^{-9}$	$9.084\times 10^{-9}$	$9.084\times 10^{-9}$
LS-EpSin	$7.432\times 10^{-6}$	$3.866\times 10^{-10}$	$1.000\times 10^{0}$	$2.619\times 10^{-6}$	$1.000\times 10^{0}$	$7.016\times 10^{-6}$
MPA	$9.084\times 10^{-9}$	$9.084\times 10^{-9}$	$9.084\times 10^{-9}$	$9.084\times 10^{-9}$	$9.084\times 10^{-9}$	$9.084\times 10^{-9}$
AHA	$9.084\times 10^{-9}$	$9.084\times 10^{-9}$	$6.025\times 10^{-6}$	$2.239\times 10^{-8}$	$9.084\times 10^{-9}$	$7.174\times 10^{-3}$
VPPSO	$3.961\times 10^{-8}$	$9.084\times 10^{-9}$	$8.315\times 10^{-7}$	$9.084\times 10^{-9}$	$9.084\times 10^{-9}$	$9.084\times 10^{-9}$
KOA	$9.084\times 10^{-9}$	$9.084\times 10^{-9}$	$9.084\times 10^{-9}$	$9.084\times 10^{-9}$	$9.084\times 10^{-9}$	$9.084\times 10^{-9}$
EAO	$1.000\times 10^{0}$	$3.866\times 10^{-10}$	$2.980\times 10^{-7}$	$3.241\times 10^{-5}$	$1.000\times 10^{0}$	$1.277\times 10^{-5}$
SFOA	$9.084\times 10^{-9}$	$9.084\times 10^{-9}$	$8.569\times 10^{-9}$	$9.084\times 10^{-9}$	$9.084\times 10^{-9}$	$9.084\times 10^{-9}$
Algorithm	F14	F15	F16	F17	F18	F19
PSO	$9.084\times 10^{-9}$	$9.084\times 10^{-9}$	$9.084\times 10^{-9}$	$9.084\times 10^{-9}$	$9.084\times 10^{-9}$	$9.084\times 10^{-9}$
LSHADE	$3.158\times 10^{-8}$	$1.981\times 10^{-5}$	$5.462\times 10^{-7}$	$3.529\times 10^{-6}$	$1.904\times 10^{-8}$	$2.242\times 10^{-2}$
GWO	$9.084\times 10^{-9}$	$9.084\times 10^{-9}$	$9.084\times 10^{-9}$	$9.901\times 10^{-9}$	$9.084\times 10^{-9}$	$9.084\times 10^{-9}$
WOA	$9.084\times 10^{-9}$	$9.084\times 10^{-9}$	$9.084\times 10^{-9}$	$9.084\times 10^{-9}$	$9.084\times 10^{-9}$	$9.084\times 10^{-9}$
LS-EpSin	$1.501\times 10^{-1}$	$1.187\times 10^{-8}$	$1.000\times 10^{0}$	$2.294\times 10^{-6}$	$1.000\times 10^{0}$	$5.679\times 10^{-6}$
MPA	$9.084\times 10^{-9}$	$8.032\times 10^{-1}$	$1.606\times 10^{-1}$	$1.000\times 10^{0}$	$3.767\times 10^{-1}$	$1.000\times 10^{0}$
AHA	$6.343\times 10^{-3}$	$6.488\times 10^{-3}$	$2.065\times 10^{-8}$	$1.140\times 10^{-3}$	$1.638\times 10^{-1}$	$7.670\times 10^{-1}$
VPPSO	$9.084\times 10^{-9}$	$9.084\times 10^{-9}$	$9.084\times 10^{-9}$	$9.084\times 10^{-9}$	$9.084\times 10^{-9}$	$9.084\times 10^{-9}$
KOA	$9.084\times 10^{-9}$	$9.084\times 10^{-9}$	$9.084\times 10^{-9}$	$9.084\times 10^{-9}$	$9.084\times 10^{-9}$	$9.084\times 10^{-9}$
EAO	$1.000\times 10^{0}$	$7.327\times 10^{-8}$	$1.170\times 10^{-2}$	$9.084\times 10^{-9}$	$1.000\times 10^{0}$	$1.366\times 10^{-5}$
SFOA	$9.084\times 10^{-9}$	$9.084\times 10^{-9}$	$9.084\times 10^{-9}$	$9.084\times 10^{-9}$	$9.084\times 10^{-9}$	$9.084\times 10^{-9}$
Algorithm	F20	F21	F22	F23	F24	F25
PSO	$9.084\times 10^{-9}$	$9.084\times 10^{-9}$	$9.084\times 10^{-9}$	$9.084\times 10^{-9}$	$3.084\times 10^{-4}$	$1.904\times 10^{-8}$
LSHADE	$2.316\times 10^{-2}$	$9.084\times 10^{-9}$	$1.000\times 10^{0}$	$2.650\times 10^{-8}$	$2.619\times 10^{-6}$	$1.981\times 10^{-5}$
GWO	$9.084\times 10^{-9}$	$9.084\times 10^{-9}$	$3.961\times 10^{-8}$	$1.366\times 10^{-5}$	$6.343\times 10^{-3}$	$2.239\times 10^{-8}$
WOA	$9.084\times 10^{-9}$	$9.084\times 10^{-9}$	$2.512\times 10^{-7}$	$9.084\times 10^{-9}$	$1.496\times 10^{-7}$	$3.696\times 10^{-8}$
LS-EpSin	$7.016\times 10^{-6}$	$7.419\times 10^{-5}$	$6.461\times 10^{-2}$	$6.989\times 10^{-5}$	$1.000\times 10^{0}$	$3.224\times 10^{-2}$
MPA	$3.734\times 10^{-5}$	$4.279\times 10^{-8}$	$1.000\times 10^{0}$	$3.126\times 10^{-1}$	$7.312\times 10^{-1}$	$1.000\times 10^{0}$
AHA	$7.430\times 10^{-2}$	$9.084\times 10^{-9}$	$1.000\times 10^{0}$	$1.228\times 10^{-4}$	$1.000\times 10^{0}$	$5.078\times 10^{-8}$
VPPSO	$9.084\times 10^{-9}$	$2.294\times 10^{-6}$	$1.193\times 10^{-5}$	$4.224\times 10^{-7}$	$1.145\times 10^{-7}$	$3.920\times 10^{-5}$
KOA	$9.084\times 10^{-9}$	$9.084\times 10^{-9}$	$9.084\times 10^{-9}$	$9.084\times 10^{-9}$	$1.215\times 10^{-2}$	$9.084\times 10^{-9}$
EAO	$8.407\times 10^{-3}$	$3.464\times 10^{-8}$	$1.000\times 10^{0}$	$1.000\times 10^{0}$	$1.000\times 10^{0}$	$1.000\times 10^{0}$
SFOA	$9.084\times 10^{-9}$	$2.905\times 10^{-9}$	$9.084\times 10^{-9}$	$8.863\times 10^{-9}$	$2.641\times 10^{-8}$	$9.084\times 10^{-9}$
Algorithm	F26	F27	F28	F29	F30	
PSO	$9.084\times 10^{-9}$	$9.084\times 10^{-9}$	$1.215\times 10^{-2}$	$9.084\times 10^{-9}$	$9.084\times 10^{-9}$	
LSHADE	$1.664\times 10^{-3}$	$4.031\times 10^{-3}$	$1.000\times 10^{0}$	$9.084\times 10^{-9}$	$3.244\times 10^{-5}$	
GWO	$4.662\times 10^{-8}$	$9.084\times 10^{-9}$	$2.168\times 10^{-2}$	$1.299\times 10^{-8}$	$9.084\times 10^{-9}$	
WOA	$4.481\times 10^{-6}$	$9.084\times 10^{-9}$	$4.031\times 10^{-3}$	$9.084\times 10^{-9}$	$9.084\times 10^{-9}$	
LS-EpSin	$3.056\times 10^{-6}$	$4.325\times 10^{-9}$	$1.000\times 10^{0}$	$3.734\times 10^{-5}$	$9.084\times 10^{-9}$	
MPA	$1.831\times 10^{-3}$	$9.084\times 10^{-9}$	$2.854\times 10^{-5}$	$1.000\times 10^{0}$	$9.084\times 10^{-9}$	
AHA	$1.000\times 10^{0}$	$9.084\times 10^{-9}$	$1.000\times 10^{0}$	$2.135\times 10^{-7}$	$9.084\times 10^{-9}$	
VPPSO	$1.000\times 10^{0}$	$9.084\times 10^{-9}$	$1.746\times 10^{-3}$	$9.084\times 10^{-9}$	$9.084\times 10^{-9}$	
KOA	$9.084\times 10^{-9}$	$9.084\times 10^{-9}$	$9.084\times 10^{-9}$	$9.084\times 10^{-9}$	$9.084\times 10^{-9}$	
EAO	$2.325\times 10^{-6}$	$3.909\times 10^{-9}$	$2.749\times 10^{-9}$	$1.000\times 10^{0}$	$9.084\times 10^{-9}$	
SFOA	$9.084\times 10^{-9}$	$8.863\times 10^{-9}$	$9.084\times 10^{-9}$	$9.006\times 10^{-9}$	$9.860\times 10^{-10}$	

**Table 8:** Suite-level Friedman ordering on CEC2022: D=10, 12 functions, and 30 runs per function.

Algorithm	Mean Rank	Final Rank
**WSAO**	**2.33**	**1**
EAO	2.54	2
LSHADE-EpSin	2.71	3
MPA	4.42	4
LSHADE	4.67	5
VPPSO	6.58	6
GWO	7.00	7
PSO	8.50	8
AHA	8.83	9
WOA	8.92	10
KOA	10.50	11
SFOA	11.00	12

**Table 9:** Complete CEC2022 results at D=10 over 30 independent runs. The best (rank-1) Avg values for each function and their corresponding Std and Rank entries are shown in bold; the proposed method name is also bold.

**Algorithm**	F1			F2			F3			F4		
	Avg	Std	Rank	Avg	Std	Rank	Avg	Std	Rank	Avg	Std	Rank
**WSAO**	**300.000**	$3.10\times 10^{-8}$	**1**	**400.507**	**1.22**	**1**	600.007	$3.63\times 10^{-3}$	4	806.339	2.57	2
PSO	597.914	$2.63\times 10^{2}$	6	456.079	47.13	9	621.163	$7.89\times 10^{0}$	9	820.713	9.65	6
LSHADE	3531.381	$4.51\times 10^{3}$	8	405.738	1.82	4	600.001	$1.62\times 10^{-3}$	3	826.265	9.87	8
GWO	741.462	$9.48\times 10^{2}$	7	414.073	13.32	7	600.533	$7.29\times 10^{-1}$	5	814.863	6.68	4
WOA	$1.75\times 10^{4}$	$7.95\times 10^{3}$	10	446.577	57.66	8	627.154	$1.58\times 10^{1}$	10	833.277	14.43	10
LS-EpSin	**300.000**	$0.00\times 10^{0}$	**1**	406.647	2.73	5	**600.000**	$1.95\times 10^{-10}$	**1**	**803.486**	**1.02**	**1**
MPA	300.136	$7.41\times 10^{-2}$	4	402.229	1.41	2	604.194	$8.33\times 10^{-1}$	6	823.963	4.68	7
AHA	$2.17\times 10^{6}$	$1.19\times 10^{7}$	12	722.985	1674.84	12	607.696	$1.92\times 10^{1}$	8	830.447	13.87	9
VPPSO	316.324	$3.45\times 10^{1}$	5	410.930	16.79	6	606.022	$7.01\times 10^{0}$	7	820.223	5.93	5
KOA	$1.07\times 10^{4}$	$2.47\times 10^{3}$	9	694.147	92.37	11	644.612	$6.15\times 10^{0}$	11	865.843	5.47	11
EAO	**300.000**	$0.00\times 10^{0}$	**1**	405.294	3.32	3	**600.000**	$1.11\times 10^{-5}$	**1**	808.720	6.77	3
SFOA	$2.07\times 10^{4}$	$1.11\times 10^{4}$	11	610.684	139.44	10	647.165	$1.66\times 10^{1}$	12	870.637	15.91	12
**Algorithm**	F5			F6			F7			F8		
	Avg	Std	Rank	Avg	Std	Rank	Avg	Std	Rank	Avg	Std	Rank
**WSAO**	**900.000**	$2.95\times 10^{-7}$	**1**	1805.180	$1.87\times 10^{0}$	3	2003.564	4.61	2	2209.644	7.52	2
PSO	998.505	$5.71\times 10^{1}$	8	2556.638	$1.20\times 10^{3}$	5	2046.028	21.71	9	2230.819	21.46	9
LSHADE	900.016	$5.03\times 10^{-2}$	4	4373.095	$4.55\times 10^{3}$	7	2018.973	6.98	5	2220.187	4.87	6
GWO	901.120	$6.63\times 10^{-1}$	5	5380.506	$2.47\times 10^{3}$	9	2026.831	8.31	7	2223.265	7.62	7
WOA	1452.309	$5.09\times 10^{2}$	10	3857.767	$1.88\times 10^{3}$	6	2064.852	21.92	10	2234.679	5.18	10
LS-EpSin	**900.000**	$0.00\times 10^{0}$	**1**	1802.000	$1.98\times 10^{0}$	2	**2002.258**	**5.44**	**1**	**2206.976**	**8.15**	**1**
MPA	915.768	$5.41\times 10^{0}$	7	1805.578	$1.27\times 10^{0}$	4	2026.792	2.89	6	2217.813	3.66	4
AHA	1275.902	$3.10\times 10^{2}$	9	$1.07\times 10^{8}$	$5.83\times 10^{8}$	12	2010.610	16.02	3	2217.934	7.43	5
VPPSO	907.671	$8.18\times 10^{0}$	6	4445.179	$2.31\times 10^{3}$	8	2040.086	15.83	8	2223.955	3.89	8
KOA	1853.587	$1.92\times 10^{2}$	11	$2.00\times 10^{7}$	$1.57\times 10^{7}$	11	2085.863	12.46	11	2244.718	6.14	11
EAO	**900.000**	$0.00\times 10^{0}$	**1**	**1800.393**	$4.88\times 10^{-1}$	**1**	2013.050	10.00	4	2212.664	7.85	3
SFOA	2087.050	$9.30\times 10^{2}$	12	$1.50\times 10^{7}$	$3.19\times 10^{7}$	10	2100.746	30.41	12	2245.884	15.34	12
**Algorithm**	F9			F10			F11			F12		
	Avg	Std	Rank	Avg	Std	Rank	Avg	Std	Rank	Avg	Std	Rank
**WSAO**	**2485.502**	$6.81\times 10^{-5}$	**1**	2503.817	19.39	4	2600.001	$5.05\times 10^{-4}$	3	**2848.024**	**9.82**	**1**
PSO	2596.088	$4.61\times 10^{1}$	10	2547.569	61.72	12	2794.617	$1.55\times 10^{2}$	7	2941.891	52.46	12
LSHADE	2485.597	$5.12\times 10^{-1}$	2	2500.423	0.08	2	2612.854	$3.73\times 10^{1}$	5	2849.649	7.79	2
GWO	2545.551	$3.28\times 10^{1}$	8	2541.600	55.17	11	2818.408	$2.01\times 10^{2}$	8	2864.110	1.33	6
WOA	2575.535	$4.70\times 10^{1}$	9	2521.920	48.08	6	2833.939	$1.75\times 10^{2}$	9	2876.568	18.85	9
LS-EpSin	2529.284	$0.00\times 10^{0}$	4	2504.203	15.31	5	**2600.000**	$4.05\times 10^{-13}$	**1**	2864.664	0.71	7
MPA	2528.405	$3.36\times 10^{0}$	3	2500.479	0.08	3	2604.671	$1.27\times 10^{0}$	4	2860.195	0.46	3
AHA	$2.54\times 10^{3}$	$1.71\times 10^{1}$	6	2538.321	64.73	10	2874.508	$5.41\times 10^{2}$	10	2913.198	212.15	10
VPPSO	2540.502	$3.69\times 10^{1}$	7	2528.123	50.80	8	2724.485	$1.78\times 10^{2}$	6	2863.503	1.95	5
KOA	2668.297	$2.71\times 10^{1}$	11	2523.142	6.55	7	2990.742	$6.61\times 10^{1}$	11	2923.781	14.06	11
EAO	2529.284	$0.00\times 10^{0}$	4	**2500.219**	**0.04**	**1**	**2600.000**	$3.48\times 10^{-13}$	**1**	2860.472	1.58	4
SFOA	2679.277	$6.66\times 10^{1}$	12	2536.943	55.57	9	3060.391	$3.32\times 10^{2}$	12	2876.452	7.63	8

**Table 10:** Holm-adjusted two-sided Wilcoxon rank-sum test *p*-values comparing WSAO with each competitor on CEC2022 at D=10. CEC2017 and CEC2022 were treated as separate test families; bold values indicate $p_{\mathrm{adj}}<0.05$.

Algorithm	F1	F2	F3	F4	F5	F6
PSO	$3.712\times 10^{-9}$	$3.712\times 10^{-9}$	$3.712\times 10^{-9}$	$1.579\times 10^{-8}$	$3.712\times 10^{-9}$	$3.712\times 10^{-9}$
LSHADE	$3.712\times 10^{-9}$	$1.120\times 10^{-8}$	$2.341\times 10^{-8}$	$8.200\times 10^{-9}$	$2.734\times 10^{-2}$	$1.150\times 10^{-6}$
GWO	$3.712\times 10^{-9}$	$3.712\times 10^{-9}$	$3.712\times 10^{-9}$	$4.674\times 10^{-7}$	$3.712\times 10^{-9}$	$3.712\times 10^{-9}$
WOA	$3.712\times 10^{-9}$	$3.950\times 10^{-9}$	$3.712\times 10^{-9}$	$3.712\times 10^{-9}$	$3.712\times 10^{-9}$	$3.712\times 10^{-9}$
LS-EpSin	$1.600\times 10^{-10}$	$2.628\times 10^{-7}$	$3.712\times 10^{-9}$	$2.499\times 10^{-4}$	$1.600\times 10^{-10}$	$1.632\times 10^{-5}$
MPA	$3.712\times 10^{-9}$	$2.880\times 10^{-6}$	$3.712\times 10^{-9}$	$3.712\times 10^{-9}$	$3.712\times 10^{-9}$	$1.000\times 10^{0}$
AHA	$3.712\times 10^{-9}$	$2.499\times 10^{-4}$	$3.049\times 10^{-1}$	$3.712\times 10^{-9}$	$3.712\times 10^{-9}$	$2.829\times 10^{-5}$
VPPSO	$4.636\times 10^{-1}$	$7.115\times 10^{-8}$	$3.712\times 10^{-9}$	$8.711\times 10^{-9}$	$7.115\times 10^{-8}$	$3.712\times 10^{-9}$
KOA	$3.712\times 10^{-9}$	$3.712\times 10^{-9}$	$3.712\times 10^{-9}$	$3.712\times 10^{-9}$	$3.712\times 10^{-9}$	$3.712\times 10^{-9}$
EAO	$1.600\times 10^{-10}$	$1.720\times 10^{-3}$	$3.289\times 10^{-9}$	$1.000\times 10^{0}$	$1.600\times 10^{-10}$	$3.712\times 10^{-9}$
SFOA	$3.712\times 10^{-9}$	$3.712\times 10^{-9}$	$3.712\times 10^{-9}$	$3.712\times 10^{-9}$	$3.712\times 10^{-9}$	$3.712\times 10^{-9}$
Algorithm	F7	F8	F9	F10	F11	F12
PSO	$3.712\times 10^{-9}$	$5.654\times 10^{-9}$	$3.712\times 10^{-9}$	$1.037\times 10^{-8}$	$3.712\times 10^{-9}$	$3.712\times 10^{-9}$
LSHADE	$1.154\times 10^{-7}$	$1.885\times 10^{-5}$	$3.049\times 10^{-1}$	$1.150\times 10^{-6}$	$1.000\times 10^{0}$	$1.000\times 10^{0}$
GWO	$4.286\times 10^{-9}$	$7.235\times 10^{-6}$	$3.712\times 10^{-9}$	$2.086\times 10^{-7}$	$3.712\times 10^{-9}$	$2.341\times 10^{-8}$
WOA	$3.712\times 10^{-9}$	$3.712\times 10^{-9}$	$3.712\times 10^{-9}$	$1.702\times 10^{-8}$	$3.712\times 10^{-9}$	$1.932\times 10^{-8}$
LS-EpSin	$2.574\times 10^{-4}$	$1.042\times 10^{-1}$	$1.600\times 10^{-10}$	$3.049\times 10^{-1}$	$9.543\times 10^{-10}$	$1.791\times 10^{-8}$
MPA	$3.712\times 10^{-9}$	$1.720\times 10^{-3}$	$3.712\times 10^{-9}$	$3.610\times 10^{-8}$	$3.712\times 10^{-9}$	$2.341\times 10^{-8}$
AHA	$1.000\times 10^{0}$	$5.755\times 10^{-2}$	$3.712\times 10^{-9}$	$1.632\times 10^{-5}$	$3.712\times 10^{-9}$	$1.932\times 10^{-8}$
VPPSO	$1.702\times 10^{-8}$	$6.101\times 10^{-8}$	$3.712\times 10^{-9}$	$1.465\times 10^{-8}$	$9.978\times 10^{-2}$	$2.341\times 10^{-8}$
KOA	$3.712\times 10^{-9}$	$3.712\times 10^{-9}$	$3.712\times 10^{-9}$	$2.341\times 10^{-8}$	$3.712\times 10^{-9}$	$3.712\times 10^{-9}$
EAO	$2.520\times 10^{-2}$	$2.416\times 10^{-1}$	$1.600\times 10^{-10}$	$1.069\times 10^{-4}$	$1.757\times 10^{-9}$	$2.291\times 10^{-8}$
SFOA	$3.712\times 10^{-9}$	$3.712\times 10^{-9}$	$3.712\times 10^{-9}$	$8.200\times 10^{-9}$	$3.712\times 10^{-9}$	$2.094\times 10^{-8}$

**Table 11:** Best-design variables and 30-run statistics for the welded-beam case. Bold rows denote algorithms tied at Rank 1 by Avg.

Algorithm	$x_{1}$	$x_{2}$	$x_{3}$	$x_{4}$	Best	Avg	Std	Rank
**WSAO**	**0.205730**	**3.253130**	**9.036790**	**0.205720**	**1.695217**	**1.695217**	$7.36\times 10^{-8}$	**1**
PSO	0.205730	3.253130	9.036790	0.205720	1.695217	1.701814	$1.94\times 10^{-2}$	8
LSHADE	0.205730	3.253130	9.036790	0.205720	1.695217	1.695230	$4.92\times 10^{-5}$	5
GWO	0.205430	3.261270	9.038780	0.205710	1.696152	1.697991	$1.53\times 10^{-3}$	6
WOA	0.186870	3.756130	8.938390	0.210280	1.750494	2.006531	$2.62\times 10^{-1}$	11
LSHADE-EpSin	0.205730	3.253130	9.036790	0.205720	1.695217	1.699504	$7.15\times 10^{-3}$	7
MPA	0.205730	3.253120	9.036790	0.205720	1.695217	1.695218	$1.25\times 10^{-6}$	4
**AHA**	**0.205730**	**3.253130**	**9.036790**	**0.205720**	**1.695217**	**1.695217**	$5.99\times 10^{-8}$	**1**
VPPSO	0.197160	3.412430	9.037560	0.205720	1.704012	1.771654	$3.73\times 10^{-2}$	9
KOA	0.199730	3.441110	9.325040	0.207830	1.777848	2.088416	$1.41\times 10^{-1}$	12
**EAO**	**0.205730**	**3.253130**	**9.036790**	**0.205720**	**1.695217**	**1.695217**	$1.13\times 10^{-15}$	**1**
SFOA	0.204070	3.287360	9.024250	0.206880	1.703955	1.883486	$1.54\times 10^{-1}$	10

**Table 12:** Best-design variables and 30-run statistics for the tension/compression-spring case. Bold rows denote algorithms tied at Rank 1 by Avg.

Algorithm	$x_{1}$	$x_{2}$	$x_{3}$	Best	Avg	Std	Rank
**WSAO**	**0.051685**	**0.356683**	**11.288406**	**0.01266346**	**0.01266346**	$2.67\times 10^{-10}$	**1**
**PSO**	**0.051685**	**0.356681**	**11.288568**	**0.01266346**	**0.01266346**	$3.81\times 10^{-18}$	**1**
LSHADE	0.051685	0.356681	11.288568	0.01266346	0.01266417	$2.75\times 10^{-6}$	7
GWO	0.051146	0.343815	12.086925	0.01266989	0.01270771	$1.80\times 10^{-5}$	8
WOA	0.051191	0.344907	12.013793	0.01266795	0.01377416	$1.32\times 10^{-3}$	11
LSHADE-EpSin	0.051685	0.356681	11.288568	0.01266346	0.01266412	$1.71\times 10^{-6}$	6
**MPA**	**0.051685**	**0.356682**	**11.288506**	**0.01266346**	**0.01266346**	$2.58\times 10^{-11}$	**1**
AHA	0.051685	0.356664	11.289554	0.01266346	0.01266364	$4.67\times 10^{-7}$	5
VPPSO	0.053138	0.392643	9.454286	0.01270107	0.01339786	$1.09\times 10^{-3}$	10
KOA	0.050279	0.322898	13.941482	0.01301292	0.01566995	$2.10\times 10^{-3}$	12
**EAO**	**0.051685**	**0.356681**	**11.288568**	**0.01266346**	**0.01266346**	$3.57\times 10^{-18}$	**1**
SFOA	0.050089	0.319664	13.825445	0.01275647	0.01316871	$9.30\times 10^{-4}$	9

**Table 13:** Best-design variables and 30-run statistics for the pressure-vessel case. The bold row denotes the Rank 1 algorithm by Avg.

Algorithm	$x_{1}$	$x_{2}$	$x_{3}$	$x_{4}$	Best	Avg	Std	Rank
WSAO	12.450698	6.154387	40.319619	199.999997	5885.332788	5885.333222	$4.13\times 10^{-4}$	2
PSO	12.558549	6.207697	40.668876	195.194557	5896.954876	6225.840866	$2.87\times 10^{2}$	8
LSHADE	12.450698	6.154387	40.319619	200.000000	5885.332775	5886.936261	$5.27\times 10^{0}$	5
GWO	12.467670	6.166110	40.374118	199.278803	5888.590306	5902.557708	$2.88\times 10^{1}$	6
WOA	13.597868	6.987809	43.891409	155.617279	6093.755068	7071.660360	$1.01\times 10^{3}$	10
LSHADE-EpSin	12.523355	6.190917	40.552373	196.821639	5894.384759	5938.560497	$2.65\times 10^{1}$	7
MPA	12.450699	6.154389	40.319619	200.000000	5885.333563	5885.341933	$1.18\times 10^{-2}$	3
AHA	12.450942	6.154655	40.320375	199.989722	5885.395428	5885.649715	$2.01\times 10^{-1}$	4
VPPSO	12.454787	6.156429	40.332859	199.815808	5885.775357	6289.130970	$2.95\times 10^{2}$	9
KOA	13.534350	7.704400	43.466259	193.003887	7088.714313	8291.042287	$8.09\times 10^{2}$	11
**EAO**	**12.450698**	**6.154387**	**40.319619**	**200.000000**	**5885.332774**	**5885.332774**	$9.25\times 10^{-13}$	**1**
SFOA	13.091826	6.371544	41.229905	200.000000	6374.727194	15781.635746	$8.97\times 10^{3}$	12

**Table 14:** Best-design variables and 30-run statistics for the three-bar-truss case. Bold rows denote algorithms tied at Rank 1 by Avg.

Algorithm	$x_{1}$	$x_{2}$	Best	Avg	Std	Rank
**WSAO**	**0.788675**	**0.408248**	**263.895843**	**263.895843**	$2.05\times 10^{-11}$	**1**
**PSO**	**0.788675**	**0.408248**	**263.895843**	**263.895843**	$2.79\times 10^{-14}$	**1**
LSHADE	0.788675	0.408248	263.895843	263.895843	$5.28\times 10^{-9}$	6
GWO	0.788190	0.409623	263.896019	263.898347	$2.01\times 10^{-3}$	8
WOA	0.788304	0.409298	263.895945	264.218971	$5.42\times 10^{-1}$	12
**LSHADE-EpSin**	**0.788675**	**0.408248**	**263.895843**	**263.895843**	$3.66\times 10^{-14}$	**1**
MPA	0.788666	0.408274	263.895844	263.896176	$8.01\times 10^{-4}$	7
AHA	0.788675	0.408248	263.895843	263.895843	$6.61\times 10^{-10}$	5
VPPSO	0.789107	0.407028	263.896021	263.901334	$5.98\times 10^{-3}$	9
KOA	0.791821	0.399473	263.908280	264.035954	$9.23\times 10^{-2}$	11
**EAO**	**0.788675**	**0.408248**	**263.895843**	**263.895843**	$0.00\times 10^{0}$	**1**
SFOA	0.786823	0.413514	263.898482	263.944289	$1.32\times 10^{-1}$	10

**Table 15:** Best-design variables and 30-run statistics for the cantilever-beam case. Bold rows denote algorithms tied at Rank 1 by Avg.

Algorithm	$x_{1}$	$x_{2}$	$x_{3}$	$x_{4}$	$x_{5}$	Best	Avg	Std	Rank
**WSAO**	**6.015571**	**5.309530**	**4.494801**	**3.501669**	**2.152090**	**1.339956**	**1.339956**	$2.56\times 10^{-8}$	**1**
PSO	6.015756	5.309027	4.494484	3.501758	2.152634	1.339956	1.339971	$3.97\times 10^{-5}$	5
LSHADE	6.016076	5.309258	4.494244	3.501363	2.152718	1.339956	1.339978	$2.69\times 10^{-5}$	6
GWO	6.004080	5.309613	4.494731	3.511260	2.154109	1.339965	1.340014	$3.58\times 10^{-5}$	8
WOA	5.668144	5.584838	4.536530	3.290546	2.668191	1.357091	1.433525	$5.58\times 10^{-2}$	10
LSHADE-EpSin	6.016018	5.309164	4.494331	3.501474	2.152672	1.339956	1.350518	$5.58\times 10^{-3}$	9
**MPA**	**6.015683**	**5.309011**	**4.494419**	**3.501720**	**2.152825**	**1.339956**	**1.339956**	$3.45\times 10^{-8}$	**1**
AHA	6.015008	5.307874	4.495474	3.502074	2.153232	1.339956	1.339957	$1.46\times 10^{-6}$	4
VPPSO	6.014604	5.314691	4.496048	3.494092	2.154273	1.339959	1.339983	$2.28\times 10^{-5}$	7
KOA	7.565456	4.948085	13.681701	5.707494	1.537663	2.086681	2.792050	$4.31\times 10^{-1}$	11
**EAO**	**6.016016**	**5.309174**	**4.494330**	**3.501475**	**2.152665**	**1.339956**	**1.339956**	$4.52\times 10^{-16}$	**1**
SFOA	5.708119	4.728801	6.999903	5.651832	1.729240	1.548637	3.696126	$1.49\times 10^{0}$	12

## Data Availability

The WSAO source code, archived benchmark results, targeted CEC2022 sensitivity and ablation package, and Holm multiple-comparison data and scripts are publicly available at https://github.com/xiaxiubo/WSAO-Weather-State-Ants-Optimizer (accessed on 21 July 2026). Release v1.1.0 contains the revision materials; tag WSAO preserves the implementation associated with the archived benchmark tables.
